# Monthly precipitation prediction based on quadratic decomposition and improved parrot algorithm

**DOI:** 10.1038/s41598-025-12493-7

**Published:** 2025-07-21

**Authors:** Weijie Zhang, Yuming Zeng, Shubo Zhou, Libin Zhang, Haiquan Li, Zhongsheng Yao, Rusheng Zhou

**Affiliations:** 1 School of Mechanical and Energy Engineering, Guangdong Ocean University, Yangjiang, 529500 China; 2 School of Computer Science and Engineering, Guangdong Ocean University, Yangjiang, 529500 China

**Keywords:** Monthly precipitation, TVMD, IPO, BiLSTM, Temporal features, Atmospheric science, Climate change

## Abstract

The amount of precipitation directly affects the ecological balance and the economic benefits of the region. However, the highly nonlinear and stochastic nature of precipitation time series data limits the accuracy of predictions. Therefore, improving the prediction accuracy of regional precipitation is crucial for formulating disaster prevention and mitigation measures, as well as for responding to climate change. To achieve a scientific and effective prediction of regional precipitation, this study proposed a precipitation prediction model based on the CEEMDAN-TVMD-IPO-BiLSTM framework. The model first decomposed the original precipitation data using the CEEMDAN decomposition algorithm, output the modal components and residual components, and then used the topology optimization algorithm (TTAO) to optimize the VMD, and decomposed the high-dimensional sequence in the first decomposition result for the second time. An improved parrot optimizer (IPO) algorithm based on chaotic Cat and Cauchy-Gaussian variation was introduced to optimize the bidirectional long short-term memory neural network (BiLSTM). This precisely constructed prediction model was utilized to predict regional precipitation, with historical monthly precipitation data from three representative cities in China—Guangzhou in the east region, Changsha in the central region, and Emeishan in the west region—used to validate the model’s accuracy and robustness. Experimental results indicated that the proposed CEEMDAN-TVMD-IPO-BiLSTM model achieved RMSE values of 32.373, 14.445, and 22.447 for the three cities, respectively, with corresponding R² values of 0.960, 0.972, and 0.977, outperforming other models. This demonstrated its advantages in monthly precipitation prediction, allowing for a better characterization of precipitation fluctuation patterns and providing scientific references for formulating policies to combat droughts and floods.

## Introduction

Intense precipitation during the summer season is a common meteorological phenomenon in the southeastern region of China^1^. The impact of climate change has led to an increased frequency of extreme precipitation events^2^while precipitation variations directly affect the ecological environment and socio-economic conditions of the region^3^. Excessive rainfall can trigger natural disasters such as flooding and landslides^4^while insufficient precipitation can result in water resource scarcity and land degradation. The prediction of precipitation remains a challenging task due to the complex nature of meteorological systems^5^. Precipitation time series exhibit high non-linearity and randomness, which are influenced by multiple uncertain factors such as geographical location, topographical features, and anthropogenic activities^6^. These characteristics introduce significant variability into precipitation patterns. As a result, the accuracy of precipitation prediction models is severely constrained by these inherent uncertainties. Therefore, improving the accuracy of regional precipitation forecasts is of paramount importance. Monthly precipitation forecasting plays a crucial role in contemporary water resource management and sustainable agricultural development^7^. Accurate monthly precipitation predictions can optimize agricultural water resource allocation, significantly enhance irrigation efficiency, and improve crop yield^8^. Furthermore, monthly precipitation forecasting provides essential decision-making support for addressing climate change challenges^6^. Long-term precipitation monitoring serves a vital function in water resource management and disaster prevention, enabling the assessment of spatial and temporal trends^9^. Additionally, monthly precipitation forecasting provides important support for estimating cultivated areas in irrigated agriculture, offering extended precipitation records for irrigation zone planning, thereby facilitating better estimation of irrigated areas that available water resources can sustain^10^.

In recent years, many experts and scholars have conducted extensive research on precipitation prediction, employing various methods. Currently, these methods can be mainly divided into two categories: traditional and modern precipitation prediction methods. The former primarily focuses on the influencing factors of precipitation, exploring the potential relationships between these factors and precipitation, and constructing predictive models using physical process analysis and mathematical statistics. In contrast, the latter uses a single precipitation time series as input and employs computer technology and artificial intelligence techniques to build data-driven models for precipitation prediction.

In traditional precipitation prediction research based on physical causation methods, Ma et al.^11^ established a comprehensive framework by extracting characteristic vectors including the intensity of the Pacific warm pool, the intensity of the polar front zone, changes in the geopotential height field at 100 hPa over the Tibetan Plateau, and the intensity of the westerly circulation as factor fields. They developed a physical statistical model to predict precipitation during the main flood season in the Sichuan-Chongqing region, demonstrating the importance of incorporating multiple atmospheric parameters. Building upon statistical methodologies, Sun et al.^12^ employed ordered clustering methods to establish grading indices for precipitation patterns and implemented weighted Markov chain models to predict future precipitation variations. Their approach, applied to nearly 50 years of precipitation data from a hydrological station in Shanxi Province, achieved satisfactory outcomes and highlighted the potential of statistical learning techniques in long-term precipitation analysis. Modern short-term precipitation forecasting has extensively utilized grey system theory approaches. Wang et al.^13^ proposed an innovative grey forecasting model specifically designed for precipitation processes in the Bohai Rim region. Their methodology incorporated function variation techniques to enhance the smoothness of precipitation time series, thereby significantly improving prediction accuracy and establishing a foundation for subsequent advanced modeling approaches.

With the rapid development of computer technology, neural network models have been widely employed in precipitation forecasting. Liu et al.^14^ employed long short-term memory network to predict precipitation in the Tibetan Plateau. Xu et al.^15^ applied temporal convolutional networks to predict precipitation in the Shanzhou District of Sanmenxia City, achieving favorable results. Through these approaches, researchers continue to enhance the accuracy and reliability of precipitation forecasts. Based on a thorough review of previous research, this paper notes the significant advantages of long short-term memory (LSTM) network in precipitation prediction, particularly in handling nonlinear time series. LSTM optimizes the traditional recurrent neural network by addressing the issues of gradient vanishing and gradient explosion that often occur during long sequence training. Kumar et al.^16^ had validated that the prediction accuracy of LSTM for monthly precipitation in climate-similar regions of India surpassed that of RNN. However, the application of LSTM in precipitation forecasting has certain limitations. Directly predicting a single time series may not fully account for the influence of the physical mechanisms underlying precipitation formation, making the model’s performance susceptible to the initial data sequence and the model’s inherent parameters^17^ To overcome this limitation, numerous scholars have proposed various coupling models.

Researchers commonly utilize wavelet decomposition and variational mode decomposition (VMD) methods to extract subsequences from complex time series data to capture the periodicity and patterns within the subsequence data. Nourani et al.^18^ proposed a hybrid approach combining discrete wavelet transform with the Mann-Kendall test, enhancing the confidence of hydrological process trend analysis through multi-temporal scale decomposition. Sattari et al.^19^ developed a probabilistic machine learning framework based on wavelet transform-LSTM Monte Carlo (PLSTM-WT) for daily extreme hydrological event prediction, decomposing observed streamflow data into constituent components through wavelet transformation to identify trend characteristics, which significantly outperformed conventional LSTM models in Hurricane Harvey event validation across southeastern Texas basins, demonstrating the effectiveness of wavelet decomposition techniques in enhancing probabilistic prediction accuracy for extreme events. Mohammadi et al.^20^ developed a hybrid framework integrating the GR6J-CemaNeige hydrological model with gradient boosting machine learning techniques, systematically incorporating hydrological process knowledge through signal processing techniques including Maximal Overlap Discrete Wavelet Transform (MODWT) and Multiresolution Analysis (MRA), achieving significant performance improvements in daily runoff prediction for northern Swedish catchments, particularly excelling in capturing complex snow-water processes. Jiang Xinyun^21^ proposed a joint prediction model based on complementary ensemble empirical mode decomposition (CEEMD) and long short-term memory neural network and conducted training and testing on the monthly precipitation in Changde City. The results indicated that the LSTM model optimized by CEEMD demonstrated high accuracy in monthly precipitation forecasting. Ren et al.^22^ utilized extreme-point symmetric mode decomposition (ESMD) and variational mode decomposition (VMD) to decompose the original precipitation time series. Subsequently, they applied LSTM predictions to each sub-series separately. The results indicated that the prediction model combining signal decomposition algorithms exhibited greater superiority in monthly precipitation forecasting. Wang et al.^23^ proposed a novel hybrid precipitation forecasting framework (WPD-ELM) that integrates Extreme Learning Machine (ELM) with Wavelet Packet Decomposition (WPD), wherein the Wavelet Packet Decomposition (WPD) technique is employed to preprocess the original precipitation data, and the Extreme Learning Machine is utilized to forecast the decomposed series. However, most of the aforementioned studies focus on either optimizing a single model structure or decomposing data sequences, with relatively few studies combining these approaches in the field of precipitation forecasting. Xu et al.^24^ applied the combination of CEEMDAN and VMD to monthly runoff prediction, and the results showed that after performing VMD secondary decomposition on the high-frequency subsequences of CEEMDAN, the prediction accuracy of the model was improved.

Monthly precipitation prediction represents a fundamental challenge in meteorological research with critical implications for water resource management, agricultural planning, and climate adaptation strategies. Current research approaches predominantly employ Long Short-Term Memory (LSTM) networks, which have demonstrated considerable success in extracting temporal features of precipitation patterns and modeling the complex relationships between various meteorological factors. However, these conventional models exhibit several inherent limitations that constrain their predictive performance. Traditional LSTM networks process temporal information unidirectionally, effectively capturing historical precipitation patterns but failing to account for how future precipitation trends may influence current precipitation levels. This unidirectional processing approach creates an incomplete understanding of precipitation dynamics, as meteorological systems often exhibit bidirectional temporal dependencies where future atmospheric conditions can provide valuable context for understanding present conditions. Furthermore, precipitation data are characterized by non-stationarity, significant volatility, and complex temporal correlations that traditional decomposition methods struggle to address adequately. Without effective signal decomposition, important temporal features remain embedded within noise components, limiting the model’s ability to identify and leverage meaningful precipitation patterns. Existing models also frequently encounter optimization challenges, where conventional parameter tuning approaches converge to local optima rather than achieving optimal network performance.

To address these limitations, this study introduces a comprehensive prediction framework combining Bidirectional LSTM networks with advanced signal processing and optimization techniques. The proposed approach employs Bidirectional LSTM (BiLSTM) networks to capture temporal dependencies by processing historical sequences in both forward and backward directions, enabling a more complete understanding of precipitation patterns within the available historical data. To effectively handle the complex, non-stationary nature of precipitation data, the framework implements a two-stage decomposition strategy. Initially, the Complete Ensemble Empirical Mode Decomposition with Adaptive Noise (CEEMDAN) algorithm decomposes the original precipitation time series into multiple components, separating different frequency patterns while preserving essential temporal characteristics. Subsequently, high-dimensional components undergo secondary decomposition using Variational Mode Decomposition (VMD) optimized through a Triangulation Topology Aggregation Optimizer (TTAO), ensuring comprehensive extraction of multi-scale temporal patterns. To overcome optimization limitations, an Improved Parrot Optimization (IPO) algorithm incorporating chaotic Cat mapping and Cauchy-Gaussian mutation strategies optimizes the BiLSTM network parameters. This enhanced optimization approach facilitates more robust convergence and improved predictive accuracy. The resulting CEEMDAN-TVMD-IPO-BiLSTM model integrates these components to provide enhanced monthly precipitation predicting capabilities that account for bidirectional temporal influences, multi-scale signal decomposition, and optimized network architecture. The effectiveness of this integrated framework is validated using historical monthly precipitation data from three geographically diverse Chinese cities: Guangzhou representing eastern coastal conditions, Changsha representing central continental patterns, and Emeishan representing western mountainous regions. This geographic diversity ensures comprehensive evaluation of the model’s robustness across varying climatic conditions and precipitation regimes, providing strong evidence for the framework’s broad applicability in regional precipitation prediction.

## Experimental method and principle

### CEEMDAN algorithm

The CEEMDAN algorithm^24^ evolved from the Empirical Mode Decomposition (EMD), Ensemble EMD (EEMD), and Complementary Ensemble EMD (CEEMD) algorithms. It effectively suppressed the mode-mixing phenomenon in EMD, generated reconstructed signals with lower residual noise than EEMD, and resolved the misalignment or errors caused by inconsistent decomposition results across subsequence groups in CEEMD. The theoretical steps of the CEEMDAN algorithm are as follows:

(1) ​Add Gaussian white noise of the same length to the original signal sequence $f(t)$ to be decomposed *n* times, thereby constructing *n* sequences to be decomposed, where $i=1,2,3, \cdots ,n$, This process can be represented as:

1 $${f_i}(t)=f(t)+{\varepsilon _0}{\delta _i}(t)$$

Where, ${\varepsilon _0}$ is the weight coefficient of Gaussian white noise, and ${\delta _i}(t)$ is the Gaussian white noise for the *i*th time.

(2) Decompose the above sequence ${f_i}(t)$ using the EMD algorithm to obtain the modal component $IM{F_i}(t)$. Repeat the decomposition *n* times and take the average to obtain the first modal component $IM{F_1}(t)$ and residual component $Re{s_1}(t)$ of CEEMDAN. This process can be represented as:

2 $$IM{F_1}(t)=\frac{1}{n}\sum\limits_{{i=1}}^{n} {IM{F_1}^{i}(t)} =\frac{1}{n}\sum\limits_{{i=1}}^{n} {EM{D_1}({f_i}(t))}$$

3 $$Re{s_1}(t)=f(t) - IM{F_1}(t)$$

(3) Add Gaussian white noise to the residual component obtained in the *k*-th stage after decomposition, and continue to apply EMD for further decomposition. This process can be represented as:

4 $$IM{F_k}(t)=\frac{1}{n}\sum\limits_{{i=1}}^{n} {EM{D_1}(Re{s_{k - 1}}(t)+{\varepsilon _{k - 1}}EM{D_{k - 1}}({\delta _i}(t)))}$$

5 $$Re{s_k}(t)=Re{s_{k - 1}}(t) - IM{F_k}(t)$$

(4) Repeat step (3) until the residual component becomes a monotonic signal and can no longer be decomposed, at which point the iteration ends. Ultimately, the original signal sequence is decomposed into *N* modal components and a residual component.

6 $$f(t)=\sum\limits_{{n=1}}^{N} {IM{F_n}} (t)+Res(t)$$

### VMD algorithm

VMD is an adaptive, completely non-recursive modal variation signal processing method^25^. Its fundamental principle is to decompose a signal into multiple components with fixed bandwidths, where each component corresponds to a specific frequency and amplitude within the signal. By optimizing a variational regularization function, VMD can adaptively match the optimal center frequency and limited bandwidth for each mode, thereby achieving effective separation of intrinsic mode functions (IMF), frequency domain partitioning of the signal, and obtaining effective decomposition components of the given signal^26^.

In addition to decomposing one-dimensional signals, VMD can also be extended to the decomposition of multi-dimensional signals. The research on multi-dimensional VMD aims to extract spatial and temporal features from multi-dimensional signals and apply them to fields such as image processing^27,28^ and video processing^13^providing new methods and insights for multi-dimensional data analysis.

The implementation formula is as follows:

7 $$\mathop {\hbox{min} }\limits_{{\{ {u_k},{\omega _k}\} }} \{ \sum\nolimits_{k} {\left\| {{\partial _t}[\delta (t)+\frac{j}{{\pi t}}]*{u_k}(t){e^{ - j{\omega _k}t}}} \right\|_{2}^{2}} \}$$

8 $$s.t.\sum\nolimits_{k} {{u_k}} =f(t)$$

Where, ${u_k}$ is the mode variable of the *k*_th_ decomposition, ${\omega _k}$ is the center frequency of ${u_k}$, $\delta (t)$ is the Dirac distribution, $\partial (t)$ is the mathematical operator for gradient calculation, and $f(t)$ is the input signal sequence to be decomposed.

To solve the optimal solution of the above variational model, a penalty factor $\alpha$ and Lagrange operator $\lambda$ are introduced to transform it into an unconstrained variational solution. The specific formula is as follows:

9 $$\begin{gathered} L(\{ {u_k}\} ,\{ {\omega _k}\} ,\lambda )=a\sum {\left\| {{\partial _t}[(\delta (t)+\frac{j}{{\pi t}})*{u_k}(t)]{e^{ - j{\omega _k}t}}} \right\|_{2}^{2}} \hfill \\ +\left\| {f(t) - \sum\nolimits_{k} {{u_k}(t)} } \right\|_{2}^{2}+\left\langle {\lambda (t),f(t) - \sum\nolimits_{k} {{u_k}(t)} } \right\rangle \hfill \\ \end{gathered}$$

Where, the penalty factor and Lagrange operator are intended to maintain the strictness of the constraints and ensure the accuracy of signal reconstruction. By continuously iterating using the alternate direction method of multipliers, the optimal solution of formula (9) is obtained. Therefore, the variables ${u_k}$ and ${\omega _k}$ can be updated according to formulas (10) and (11):

10 $$u_{n}^{{n+1}}=(f(t) - \sum\nolimits_{{i \ne k}} {{u_i}+\frac{\lambda }{2}} )\frac{1}{{1+2\alpha {{(\omega - {\omega _k})}^2}}}$$

11 $$\omega _{n}^{{n+1}}=\frac{{\int_{0}^{\infty } {\omega {{\left| {{u_k}(\omega )} \right|}^2}d\omega } }}{{\int_{0}^{\infty } {{{\left| {{u_k}(\omega )} \right|}^2}d\omega } }}$$

Where, *n* represents the iteration number.

### TTAO algorithm

The TTAO algorithm^29^ is based on the principle of similar triangles. During the iterative process, it searches using the three vertices and one interior point of the triangular topology unit. Optimization is achieved through aggregation within the triangular topology unit and between different triangular topology units. The optimization process consists of three main stages: the construction of triangular topology units, global aggregation, and local aggregation.

(1) Construction of triangular topology units: The number of individuals *N* can be divided into $[{N \mathord{\left/ {\vphantom {N 3}} \right. \kern-0pt} 3}]$ triangular topology units, where $[\cdot ]$ represents the floor value. The process is as follows:

12 $$\overrightarrow {{X_{i,1}}} ={r_0} \times (\overrightarrow {UB} - \overrightarrow {LB} )+\overrightarrow {LB}$$

13 $$\overrightarrow {{X_{i,2}}} =\overrightarrow {{X_{i,1}}} +l \times \overrightarrow {f(\overrightarrow \theta )}$$

14 $$\overrightarrow {{X_{i,3}}} =\overrightarrow {{X_{i,1}}} +l \times \overrightarrow {f(\overrightarrow {\theta +\frac{\pi }{3}} )}$$

15 $$\overrightarrow {{X_{i,4}}} ={r_1} \times \overrightarrow {{X_{i,1}}} +{r_2} \times \overrightarrow {{X_{i,2}}} +{r_3} \times \overrightarrow {{X_{i,3}}}$$

Where, $\overrightarrow {{X_{i,1}}}$, $\overrightarrow {{X_{i,2}}}$ and $\overrightarrow {{X_{i,3}}}$ represent the three vertices of the *i*_th_ triangular topology unit, and $\overrightarrow {{X_{i,4}}}$ indicates a randomly chosen interior vertex within the *i*_th_ triangular topology unit. $\overrightarrow {UB}$ and $\overrightarrow {LB}$ are the upper and lower bounds of the search space. *l* represents the size of the triangular topology unit. Additionally, $\overrightarrow {f\left( {\overrightarrow \theta } \right)}$ and $\overrightarrow {f\left( {\overrightarrow {\theta +\frac{\pi }{3}} } \right)}$ denote the direction vectors of the other two edges guided by the first vertex.

(2) Global aggregation: This process represents the exploration phase of the algorithm. By collecting information from excellent individuals within different triangular topology units, new feasible solutions are generated. Specifically, at *k*_th_ iteration, the optimal vertex $\overrightarrow {X_{{i,best}}^{k}}$ of each triangular topology unit interacts with the optimal vertex $\overrightarrow {X_{{rand,best}}^{k}}$ of a randomly selected topology unit to aggregate and form a new feasible solution $\overrightarrow {X_{{i,new1}}^{{k+1}}}$, as shown in the following formula:

16 $$\overrightarrow {X_{{i,new1}}^{{k+1}}} ={r_4} \times \overrightarrow {X_{{i,best}}^{k}} +(1 - {r_4}) \times \overrightarrow {X_{{rand,best}}^{k}}$$

Next, the fitness of the new solution is compared with the fitness of the optimal or suboptimal solution at *k*_th_ iteration, in order to update the optimal solution, that is:

17 $$\begin{array}{*{20}{c}} {\overrightarrow {X_{{i,best}}^{{k+1}}} =\overrightarrow {X_{{i,new1}}^{{k+1}}} }&{{f_{\overrightarrow {X_{{i,new1}}^{{k+1}}} }}<{f_{\overrightarrow {X_{{i,best}}^{k}} }}} \\ {\overrightarrow {X_{{i,sbest}}^{{k+1}}} =\overrightarrow {X_{{i,new1}}^{{k+1}}} }&{{f_{\overrightarrow {X_{{i,new1}}^{{k+1}}} }}<{f_{\overrightarrow {X_{{i,sbest}}^{k}} }}} \end{array}$$

(3) Local aggregation: This process is the development phase of the algorithm. Within each triangular topology unit, perturbations are applied to the optimal solution based on the differences between the optimal and suboptimal solutions, in order to prevent the optimal individuals from getting trapped in local optima, as shown in Eq. (18).

18 $$\overrightarrow {X_{{i,new2}}^{{k+1}}} =\overrightarrow {X_{{i,best}}^{{k+1}}} +\alpha \times (\overrightarrow {X_{{i,best}}^{{k+1}}} - \overrightarrow {X_{{i,sbest}}^{{k+1}}} )$$

Where, the $\alpha$ is continuously reduced to allow the algorithm to gradually approach the optimal solution. To improve convergence, the fitness values of the two vertices before and after the local development are compared to determine the update position. If the new individual is better than the original individual, the position is updated; otherwise, no update is performed, that is:

19 $$\overrightarrow {X_{{i,best}}^{{k+1}}} =\left\{ {\begin{array}{*{20}{c}} {\overrightarrow {X_{{i,new2}}^{{k+1}}} }&{{f_{\overrightarrow {X_{{i,new2}}^{{k+1}}} }}<{f_{\overrightarrow {X_{{i,best}}^{{k+1}}} }}} \\ {\overrightarrow {X_{{i,best}}^{{k+1}}} }&{{\text{oth}}erwise} \end{array}} \right.$$

### TVMD algorithm

The VMD algorithm improves the predictive accuracy of data by decomposing it to reduce non-stationarity and non-linearity. However, if the number of modes for decomposition is too few, it may leave behind a residual with high complexity, which cannot guarantee prediction accuracy. Conversely, if the number of modes is too high, it can lead to over-decomposition of the data. Therefore, in the VMD algorithm, the number of decomposed modes *k* and the penalty factor $\alpha$ are interdependent and jointly affect the decomposition results. Thus, obtaining the optimal parameter combination $[k,\alpha ]$ based on the characteristics of the input signal is key to achieving adaptive decomposition through VMD.

Therefore, this paper employed the TTAO optimization algorithm to optimize the optimal parameter combination of the VMD decomposition algorithm, referred to as the TVMD decomposition algorithm. This algorithm uses the minimum envelope entropy $\hbox{min} {E_e}$ as the fitness function. After the signal undergoes VMD decomposition, the more noise contained in the subsequences, the greater the envelope entropy value will be. The calculation formula for envelope entropy is as follows:

20 $$\left\{ {\begin{array}{*{20}{c}} {{E_e}= - \sum\limits_{{j=1}}^{N} {{b_j}\lg {b_j}} } \\ {{b_j}=a(j)/\sum\limits_{{j=1}}^{N} {a(j)} } \end{array}} \right.$$

Where, $a(j)$ represents the result obtained by applying Hilbert transform to the IMF component of the original data after VMD decomposition. ${b_j}$ is the normalized form of $a(j)$.

The steps of the proposed TVMD decomposition algorithm are as follows:

(1) Initialize the TTAO parameters, set the population size, number of iterations, and the value ranges for *K* and $\alpha$.

(2) Obtain the fitness value using Eq. (20), and update the positions of the triangular topology units according to Eqs. (17) and (19) until the maximum number of iterations is reached, resulting in the corresponding optimal parameter combination $[k,\alpha ]$.

(3) Perform VMD decomposition on the original precipitation data based on the optimal parameter combination, outputting *K* intrinsic mode function components and the residual component.

### PO algorithm

The Parrot Optimization Algorithm^30^ is a meta heuristic optimization algorithm proposed in 2024, which solves the optimal parameters by simulating the four key behavioral characteristics of parrots. The solution process is as follows:

(1) Population Initialization: The algorithm initializes a set of candidate solutions as the parrot population, with each parrot representing a potential solution. Assume the population size is *N*, the maximum number of iterations is $Ma{x_{iter}}$ and the lower and upper bounds of the search space are *lb* and *ub*. The initial positions of the parrots are:

21 $$X_{w}^{0}=lb+rand(0,1)\cdot (ub - lb)$$

Where, $rand(0,1)$ represents a random number in the range $[0,1]$, and $X_{w}^{0}$ denotes the initial position of the *w*_th_ parrot.

(2) Foraging behavior: Parrots estimate the approximate location of food by observing its position or the position of its owner, and then fly towards their respective positions, where the position movement follows the following equation:

22 $$X_{w}^{{d+1}}=(X_{w}^{d} - {X_{best}})\cdot Levy(dim)+rand(0,1)\cdot {(1 - \frac{t}{{Ma{x_{iter}}}})^{\frac{{2d}}{{Ma{x_{iter}}}}}}\cdot X_{{mean}}^{d}$$

Where, $X_{w}^{d}$ represents the current position, $X_{w}^{{d+1}}$ denotes the updated position, ${X_{best}}$ indicates the best position found so far and the master’s current position, $Levy(dim)$ denotes the Levy distribution, which describes the parrot’s flight, *d* indicates the current iteration number, and $X_{{mean}}^{d}$ represents the average position of the current population, i.e.:

23 $$X_{{mean}}^{d}=\frac{1}{N}\sum\limits_{{k=1}}^{N} {X_{k}^{d}}$$

(3) Staying Behavior: The parrot suddenly flies to any part of the owner’s body and remains still for a period of time. This process can be represented as:

24 $$X_{w}^{{d+1}}=X_{w}^{d}+{X_{best}}\cdot Levy(dim)+rand(0,1)\cdot ones(1,dim)$$

Where, $ones(1,dim)$ represents an all-ones vector of dimension *dim*.

(4) Communication Behavior: This behavior involves close interaction within the flock, including both flying towards and not flying towards the group. It is simulated by calculating the average position of the population and adjusting candidate solutions accordingly to promote information sharing and collaboration. This process can be represented as:

25 $$X_{w}^{{d+1}}=\left\{ {\begin{array}{*{20}{c}} {0.2rand(0,1)\cdot (1 - \frac{d}{{Ma{x_{iter}}}})\cdot (X_{w}^{d} - X_{{mean}}^{d})}&{P \leqslant 0.5} \\ {0.2rand(0,1)\cdot \exp ( - \frac{d}{{rand(0,1)Ma{x_{iter}}}})}&{P>0.5} \end{array}} \right.$$

Where, when $P \leqslant 0.5$ represents an individual joining a parrot group for communication, when $P>0.5$ represents the process of the individual immediately flying out after communication.

(5) Fear behavior: Individuals usually keep a distance from unfamiliar individuals and seek a safe environment together with their owner. This behavior avoids excessive concentration of candidate solutions through a repulsion mechanism, maintaining population diversity. This process can be represented as:

26 $$\begin{gathered} X_{w}^{{d+1}}=X_{w}^{d}+rand(0,1)\cdot \cos (0.5\pi \cdot \frac{d}{{Ma{x_{iter}}}})\cdot ({X_{best}} - X_{w}^{d}) \hfill \\ - \cos (rand(0,1)\cdot \pi )\cdot {(\frac{d}{{Ma{x_{iter}}}})^{\frac{2}{{Ma{x_{iter}}}}}}\cdot (X_{w}^{d} - {X_{best}}) \hfill \\ \end{gathered}$$

### IPO algorithm

(1) Population initialization with chaotic reverse learning strategy.

Traditional population initialization methods rely heavily on random number generation, which can lead to uneven distribution of individuals in the search population. To maintain population diversity and ensure that the initial population is as evenly distributed as possible, this paper introduced an initialization strategy based on chaotic reverse learning^31^. This approach helps accelerate the convergence speed of the algorithm. The steps of this strategy are: first, use the Cat chaotic sequence to generate *N* initial solutions ${X_i}$. For each initial solution, generate the corresponding reverse solution using the following method:

27 $$O{P_i}=rand(0,1)\cdot (X_{{\hbox{min} }}^{d}+X_{{\hbox{max} }}^{d}) - {X_i}$$

Where, $X_{{\hbox{min} }}^{d}$ and $X_{{\hbox{max} }}^{d}$ represent the minimum and maximum values of the *d*_th_ dimension vector among all initial solutions.

Finally, the initial solutions with the reverse solutions was combine to sort in ascending order based on their fitness values. The top *N* solutions with the best fitness values was selected to form the initial population.

(2) Improving Nonlinear Convergence Factor Strategy.

When $P \leqslant 0.5$ represents an individual joining a parrot group for communication, when $P>0.5$ represents the process of the individual immediately flying out after communication. Therefore, the value of *P* is closely related to the communication behavior in the parrot optimization algorithm. However, in the traditional parrot algorithm, the value of *P* is random, which does not reflect the changes in the optimization algorithm during the iterative process. Therefore, this paper proposed an improved nonlinear formula:

28 $$P=rand(0,1)\cdot \frac{{Ma{x_{iter}} - d}}{{Ma{x_{iter}}}}$$

(3) Cauchy-Gaussian variation.

The variation strategy can prevent the algorithm from getting trapped in local optima and also maintain the diversity of the population. To reduce the probability of the parrot algorithm falling into local optima, this paper introduced the Cauchy-Gaussian variation operator^32^. This mutation operator combines the Cauchy variation operator^33^ and the Gaussian variation operator^34^. It allows global search during the early stages of population optimization and local search during the later stages of iteration, thereby significantly enhancing its optimization capability. The expression for this operator is:

29 $$X_{{new}}^{d}=X_{{best}}^{d} \times [1+{\beta _1}Cauchy(0,1)+{\beta _2}Causs(0,1)]$$

Where, $$X_{{best}}^{d}$$ represents the optimal position of the parrot population in the dth iteration. $$X_{{new}}^{d}$$ is the new position generated from the optimal position in the dth iteration using the Cauchy-Gaussian variation strategy. $$Cauchy(0,1)$$ and $$Causs(0,1)$$are the random number following a Cauchy distribution and a Gaussian distribution. $${\beta _1}=1 - {\raise0.7ex\hbox{$d$} \!\mathord{\left/ {\vphantom {d {Ma{x_{iter}}}}}\right.\kern-0pt}\!\lower0.7ex\hbox{${Ma{x_{iter}}}$}}$$ and $${\beta _2}={\raise0.7ex\hbox{$d$} \!\mathord{\left/ {\vphantom {d {Ma{x_{iter}}}}}\right.\kern-0pt}\!\lower0.7ex\hbox{${Ma{x_{iter}}}$}}$$.

### BiLSTM network

The LSTM network model^35^ is proposed based on recurrent neural network^36^ and is renowned for its excellent ability to process sequential data. The LSTM model introduces gate control units (forget gate, input gate, and output gate) and memory cell states, which address the issues of gradient vanishing and gradient explosion that often occur during long sequence training^37^.

LSTM network can only encode time series data in a forward direction and cannot learn patterns from both forward and backward information in the sequence. In contrast, BiLSTM neural networks consist of a combination of forward and backward LSTM, allowing them to consider the impact of forward and backward time series data on the current state. Therefore, BiLSTM typically provides better prediction accuracy than LSTM^38^.

## Materials and methods

### Monthly precipitation prediction model

**Fig. 1:**
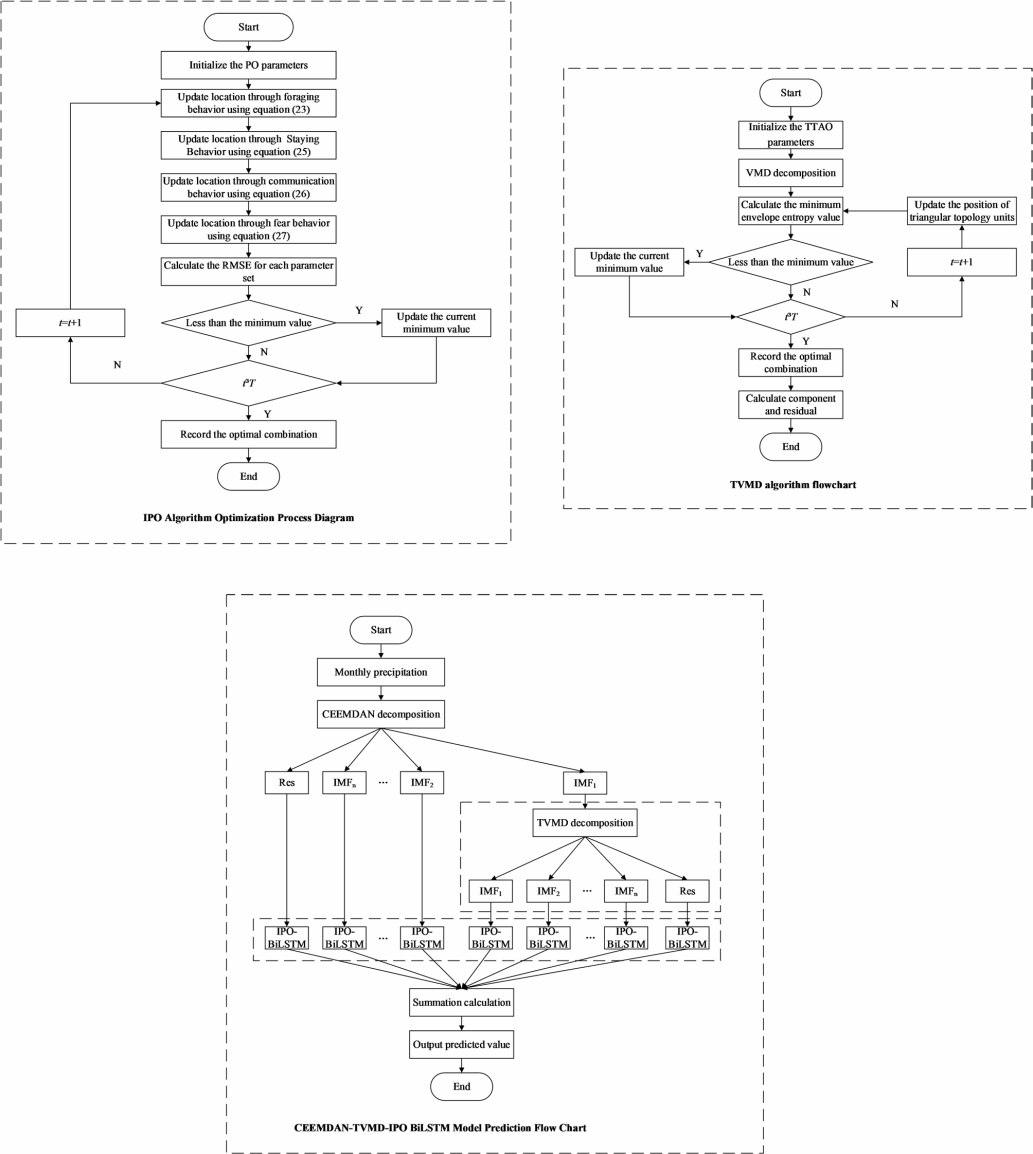
Flowchart of the Prediction Model.

To further explore the temporal features of monthly precipitation data, this paper established a precipitation prediction model based on CEEMDAN-TVMD-IPO-BiLSTM. The flowchart of the model is shown in Fig. 1, with the specific steps as follows:

(1) Using the CEEMDAN decomposition algorithm to decompose the original precipitation data, outputting intrinsic mode function components and residual components;

(2) Initialize the TTAO parameters, set the population size, number of iterations, and the value ranges for *K* and $\alpha$.

(3) Obtain the fitness value using Eq. (20), and update the positions of the triangular topology units according to Eqs. (17) and (19) until the maximum number of iterations is reached, resulting in the corresponding optimal parameter combination $[k,\alpha ]$.

(4) Perform VMD decomposition on the high-dimensional components in the first decomposition result based on the optimal parameter combination, outputting *K* intrinsic mode function components and the residual component.

(5) Use the IPO algorithm to optimize the parameters of the BiLSTM, finding the optimal parameters for each component (learning rate, number of hidden layers in BiLSTM, and number of iterations).

(6) Divide the original data into a training set and a test set, with the first 70% of the data used as the training set and the latter 30% used as the test set to evaluate the model’s prediction accuracy. To ensure robust model validation and prevent overfitting, a five-fold cross-validation strategy is implemented within the training set during the hyperparameter optimization process.

(7) Input the training set samples into the IPO-BiLSTM model to train the model, and then input the test set into the model to assess the prediction performance of the model.

(8) Finally, the prediction results of each subsequence and residual of the first and second decompositions are superimposed to obtain the final monthly precipitation prediction value. It should be noted that the entire optimization process, including TTAO parameter optimization for TVMD and IPO hyperparameter tuning for BiLSTM, is performed separately for each of the three cities (Guangzhou, Changsha, and Emeishan) to account for their distinct precipitation characteristics and local climatic conditions, ensuring location-specific model performance optimization.

### Data processing

**Fig. 2:**
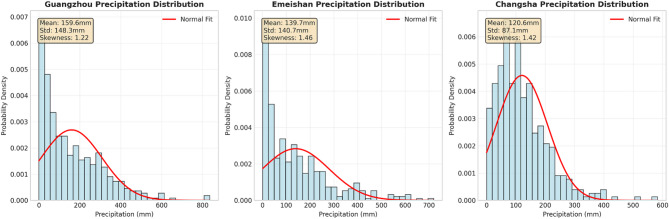
Distribution of Precipitation.

Using the “Global Surface Recompiled Dataset - Daily Products,” independently developed by the National Meteorological Information Center, as the source for monthly statistical values, the statistical monthly values are further integrated with the United States’ GHCNM to form a consolidated dataset. GHCNM, managed by the U.S. NCEI, collects massive amounts of foundational global data through long-term international exchange and data rescue efforts. Based on this, data comparison, integration, and quality control processes are gradually carried out to form an authoritative set of foundational global data products, which have been applied in the operations and research work of various international organizations and research institutes. This paper selected the monthly precipitation data from 1990 to 2022 for three cities: Guangzhou in Guangdong Province in the eastern region, Changsha in Hunan Province in the central region, and Emeishan in Sichuan Province in the western region. The training set and test set account for 70% and 30% of the entire dataset, respectively. This article uses minimum, maximum, median, mean, standard deviation (std.), coefficient of variation (cv), and skewness to statistically analyze precipitation data. The data statistics of precipitation in three cities are shown in Table 1; Fig. 2.

**Table 1:** Precipitation data statistics.

City	Minimum	Maximum	Median	Mean	Std.	CV	Skewness
Guangzhou	0	835.3	116.8	159.6	148.3	0.93	1.22
Emeishan	1.3	719.6	97.0	139.7	140.7	1.01	1.46
Changsha	0	583.1	104.0	120.6	87.1	0.72	1.42

Before inputting data into the neural network, it is necessary to perform normalization to constrain the data within a certain range, thereby accelerating the convergence process^39^. To reduce the differences between data points, this paper adopted the min-max normalization method to scale the precipitation data to the [0,1] interval. The formula for this calculation is:

30 $${x^ * }=\frac{{{x_i} - {x_{\hbox{min} }}}}{{{x_{\hbox{max} }} - {x_{\hbox{min} }}}}$$

Where, ${x_i}$ is the current precipitation value, while ${x_{\hbox{min} }}$ and ${x_{\hbox{max} }}$ are the minimum and maximum precipitation values in the dataset, respectively.

This paper used a sliding window to reconstruct the dataset. The window should be set to include as much information as possible; however, if the window length is too long, it can lead to redundant information. In this study, the window length was set to 6, and by sliding the window backward with a step size of 1, multiple time-step features were obtained, resulting in multiple sets of input-output data.

### Evaluation metric

To objectively evaluate the prediction accuracy and effectiveness of the CEEMDAN-TVMD-IPO-BiLSTM model on the dataset in this paper, the root mean square error (RMSE), mean absolute error (MAE), mean squared error (MSE), and R-squared (*R*²) were chosen to measure the model’s prediction accuracy. The Willmott index of agreement (WIA) was selected to assess the model’s external prediction ability and generalization capability. A larger WIA indicates a higher degree of match between the estimated values and actual values. The calculation formulas were provided in Eqs. (31)-(35).

31 $$RMSE=\sqrt {\frac{1}{m}\sum\limits_{{i=1}}^{m} {{{({y_i} - {{\hat {y}}_i})}^2}} }$$

32 $$MAE=\frac{1}{m}\sum\limits_{{i=1}}^{m} {\left| {{y_i} - {{\hat {y}}_i}} \right|}$$

33 $$MSE=\frac{1}{m}\sum\limits_{{i=1}}^{m} {{{({y_i} - {{\hat {y}}_i})}^2}}$$

34 $${R^2}=1 - \frac{{\sum\limits_{{i=1}}^{m} {{{({y_i} - {{\hat {y}}_i})}^2}} }}{{\sum\limits_{{i=1}}^{m} {{{({y_i} - \frac{1}{m}\sum\limits_{{i=1}}^{m} {{y_i}} )}^2}} }}$$

35 $$I(WIA)=1 - \frac{{\sum\limits_{{i=1}}^{m} {{{({y_i} - {{\hat {y}}_i})}^2}} }}{{\sum\limits_{{i=1}}^{m} {{{(\left| {{y_i} - \frac{1}{m}\sum\limits_{{i=1}}^{m} {{y_i}} } \right|+\left| {{{\hat {y}}_i} - \frac{1}{m}\sum\limits_{{i=1}}^{m} {{y_i}} } \right|)}^2}} }}$$

Where, ${y_i}$ represents the actual monthly precipitation values, ${\hat {y}_i}$ represents the predicted monthly precipitation values, and *m* is the number of predictions.

Furthermore, this study also evaluated the computational time required for the model during parameter optimization, model training, and model prediction phases, denoted as T(optimize), T(train), and T(predict), respectively, with all time units measured in seconds.

### Experimental configuration

The experiments in this study were conducted on a desktop computer equipped with an Intel(R) Core(TM) i7-10700KF CPU, 64GB RAM, and RTX3070 GPU, using MATLAB R2024b. The parameter configurations for the LSTM and BiLSTM models were set as follows: 128 hidden layer units, maximum training epochs of 500, Adam optimizer, and an initial learning rate of 0.01. The optimization parameters for PO and IPO algorithms included initial learning rate, number of hidden layer units, and maximum training epochs, where the initial learning rate ranged from 0.001 to 0.01, hidden layer units ranged from 16 to 128, and maximum training epochs ranged from 300 to 500.

## Experiment results and analysis

### Selected model result

To validate the effectiveness of the selected model, precipitation prediction accuracy was evaluated across three cities using different models. Five baseline models were selected for comparison: Back Propagation Neural Network (BP)^40^Random Forest (RF)^41^Support Vector Machine (SVM)^42^Long Short-Term Memory (LSTM)^43^and Bidirectional Long Short-Term Memory (BiLSTM)^44^. These models represented different categories of machine learning and deep learning methods, providing a comprehensive benchmark for evaluating the performance of various prediction approaches. The experimental results are presented in Table 2.

The analysis of experimental results for different models in Table 2 demonstrates that although the training time of BiLSTM and LSTM models is substantially greater than that of the three computational models, the prediction time required by these five machine learning models shows no significant differences. Moreover, the prediction accuracy of BiLSTM and LSTM models is superior to that of other models, indicating that recurrent neural networks possess superior capability in capturing temporal dependencies in precipitation data. Moreover, compared to the LSTM model, the BiLSTM model was able to process both forward and backward temporal information, enabling more comprehensive feature extraction. Traditional machine learning methods, such as BP, RF, and SVM models, exhibited lower prediction performance, indicating their limitations in simulating the inherent complex nonlinear patterns in precipitation sequences. The experimental results demonstrated that the BiLSTM model provided the most robust and accurate predictions across different geographical regions.

**Table 2:** Prediction errors of different model algorithms.

City	Model	MSE	MAE	RMSE	*R* ^2^	I(WIA)	T(train)	T(predict)
Changsha	BP	5928.680	56.968	76.998	0.194	0.651	0.576	0.005
	RF	5607.839	55.383	74.886	0.238	0.631	0.167	0.009
	SVM	6205.869	57.579	78.777	0.157	0.592	0.007	0.001
	LSTM	5577.718	53.829	74.684	0.242	0.606	29.441	0.006
	BiLSTM	5545.786	54.253	74.470	0.247	0.624	32.582	0.007
Emeishan	BP	7217.622	54.252	84.957	0.666	0.894	0.450	0.005
	RF	7334.562	51.262	85.642	0.661	0.884	0.218	0.008
	SVM	7808.780	55.681	88.367	0.639	0.868	0.012	0.001
	LSTM	7236.412	52.050	85.067	0.665	0.888	28.250	0.007
	BiLSTM	7027.275	51.087	83.829	0.675	0.895	33.357	0.007
Guangzhou	BP	19693.431	94.541	140.333	0.247	0.702	0.463	0.006
	RF	18277.982	97.433	135.196	0.301	0.679	0.157	0.010
	SVM	19279.689	93.231	138.851	0.263	0.705	0.005	0.001
	LSTM	17658.913	91.935	132.887	0.325	0.720	29.231	0.007
	BiLSTM	17375.193	91.388	131.815	0.335	0.726	32.258	0.006

### Decomposition algorithm result

To verify the accuracy and feasibility of the decomposition algorithm proposed in this paper, this section conducted comparative experiments using the CEEMDAN-TVMD-BiLSTM model, the TVMD-BiLSTM model, the CEEMDAN-BiLSTM model, the VMD-BiLSTM model, and the BiLSTM model in three typical cities. The prediction results of these models were shown in Table 3.

From the comparison results of different models in Table 3, it could be seen that after decomposing the original precipitation sequence data using the decomposition algorithm, whether it was VMD, TVMD, CEEMDAN or CEEMDAN-TVMD decomposition algorithm, they could better capture the nonlinear fluctuation patterns in different dimensions of the sequence data, greatly improving the accuracy of the prediction model. Additionally, by comparing the VMD-BiLSTM and TVMD-BiLSTM models for the cities of Guangzhou, Changsha, and Emeishan, it could be observed that the parameters optimized through TTAO allowed for a more precise adaptive decomposition of precipitation sequence data in different locations. Comparing the CEEMDAN BiLSTM model, TVMD BiLSTM model, and CEEMDAN BiLSTM model of Changsha, Emeishan and Guangzhou, it could be found that the decomposition effect of the CEEMDAN decomposition algorithm was worse than that of the TVMD decomposition algorithm, and the decomposition effect of the secondary decomposition algorithm on the data was better than that of the primary decomposition algorithm. Consequently, the model constructed in this paper achieved a more robust and accurate prediction of precipitation across various regions.

**Table 3:** Prediction errors of different decomposition algorithms.

City	Model	MSE	MAE	RMSE	*R* ^2^	I(WIA)	T(train)	T(predict)
Changsha	BiLSTM	5545.786	54.253	74.470	0.247	0.624	32.582	0.007
	VMD-BiLSTM	1832.031	32.800	42.802	0.751	0.914	231.943	0.046
	CEEMDAN-BiLSTM	2116.571	35.048	46.006	0.712	0.913	310.477	0.062
	TVMD-BiLSTM	247.037	12.149	15.717	0.966	0.991	308.711	0.061
	CEEMDAN-TVMD-BiLSTM	236.572	12.298	15.381	0.968	0.991	473.128	0.091
Emeishan	BiLSTM	7027.275	51.087	83.829	0.675	0.895	33.357	0.007
	VMD-BiLSTM	1977.555	31.719	44.470	0.909	0.974	234.731	0.045
	CEEMDAN-BiLSTM	2541.294	40.011	50.411	0.882	0.967	305.774	0.062
	TVMD-BiLSTM	898.157	21.995	29.969	0.958	0.989	345.738	0.069
	CEEMDAN-TVMD-BiLSTM	619.635	19.599	24.892	0.971	0.992	634.697	0.122
Guangzhou	BiLSTM	17375.193	91.388	131.815	0.335	0.726	32.258	0.006
	VMD-BiLSTM	4095.575	48.933	63.997	0.843	0.951	236.288	0.051
	CEEMDAN-BiLSTM	6787.323	63.939	82.385	0.740	0.921	302.326	0.062
	TVMD-BiLSTM	1824.717	32.019	42.717	0.930	0.980	341.501	0.068
	CEEMDAN-TVMD-BiLSTM	1208.064	26.606	34.757	0.954	0.987	536.747	0.110

**Fig. 3:**
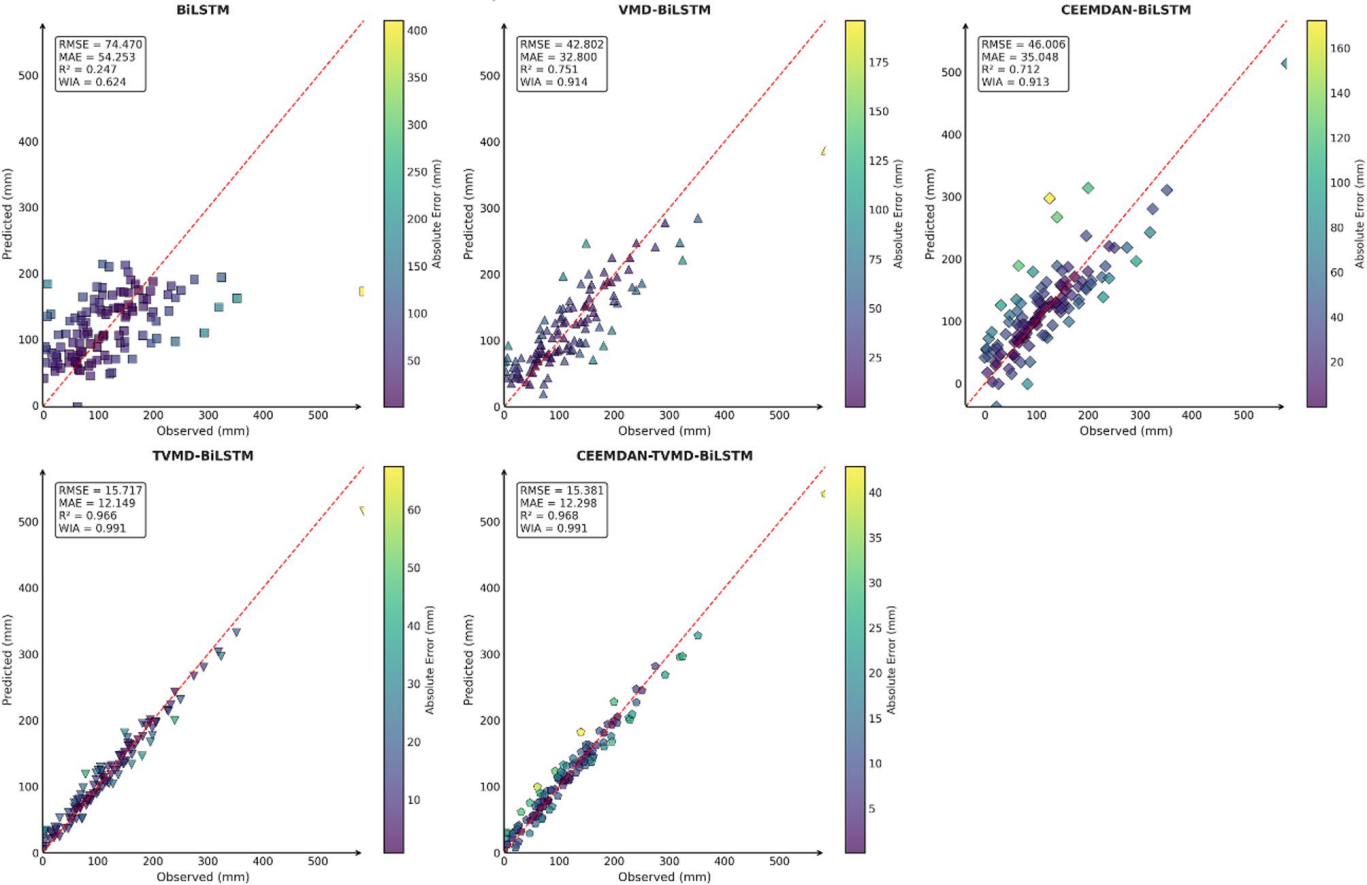
Scatter plot of predicted and observed values in Changsha.

**Fig. 4:**
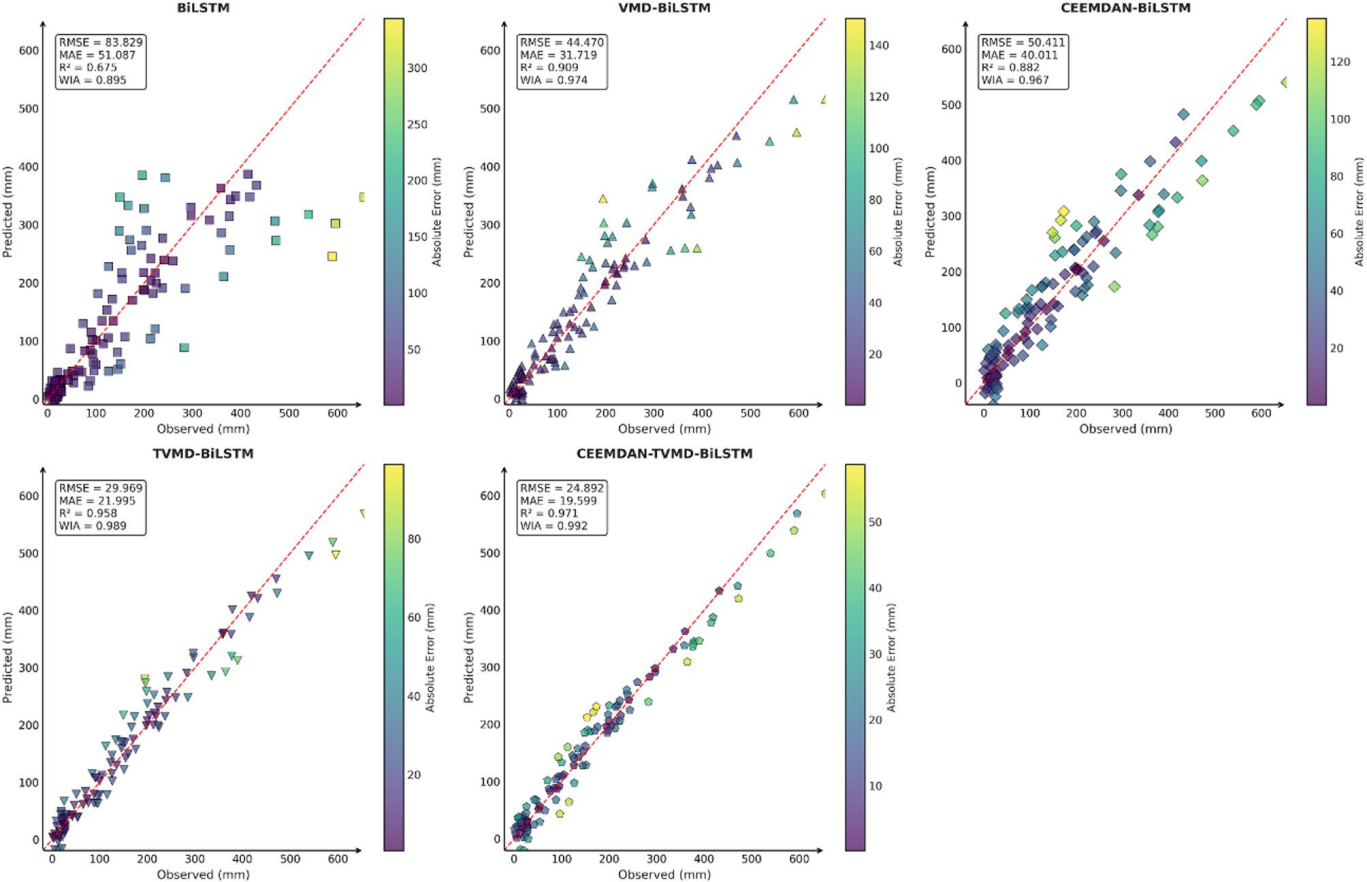
Scatter plot of predicted and observed values in Emeishan.

**Fig. 5:**
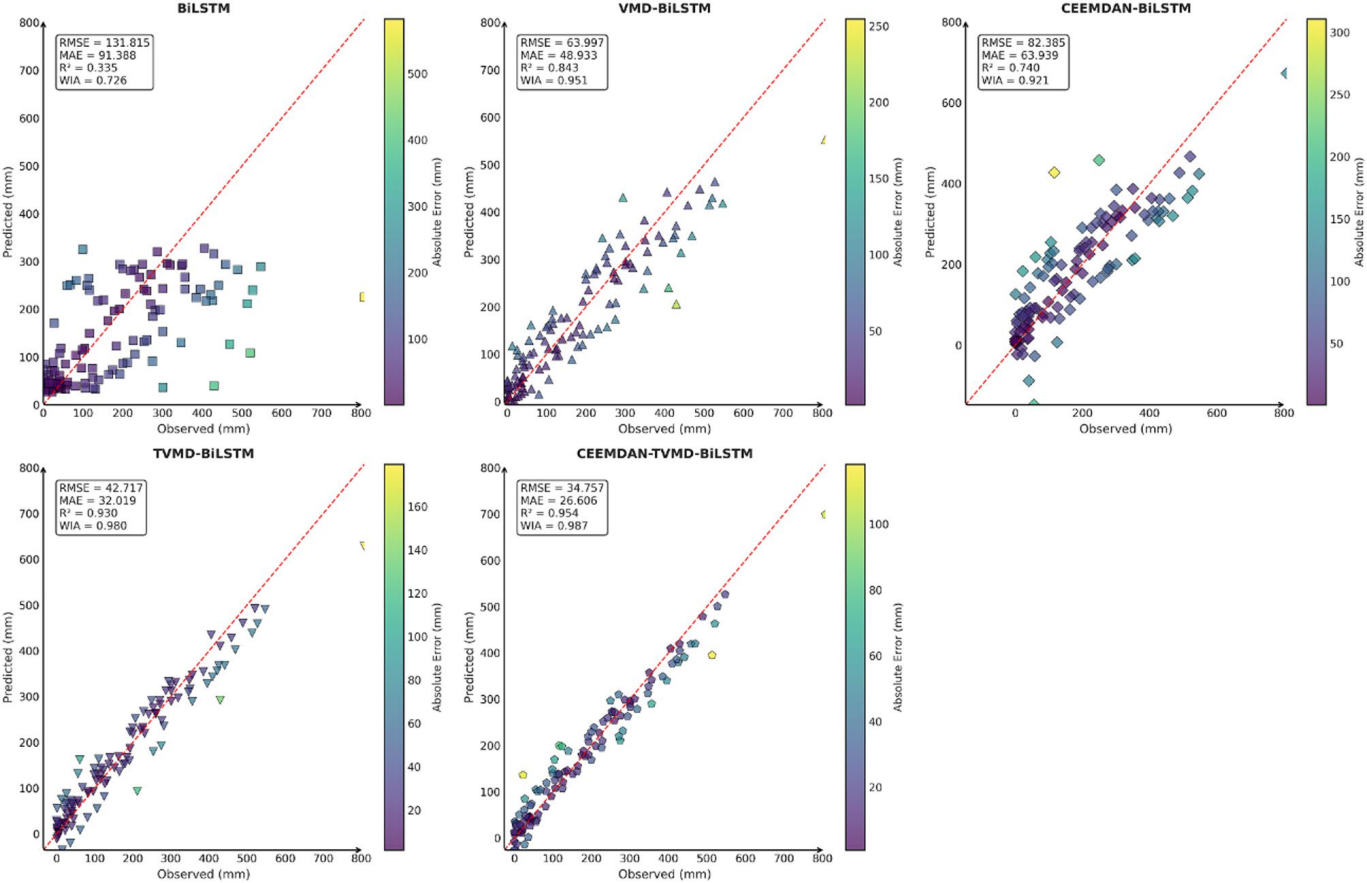
Scatter plot of predicted and observed values in Guangzhou.

**Fig. 6:**
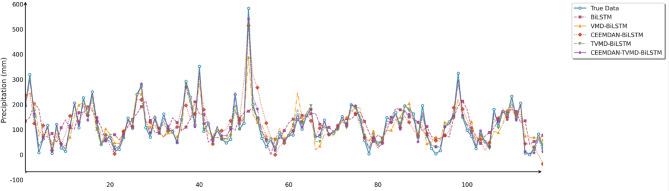
Line chart of predicted and observed values in Changsha.

**Fig. 7:**
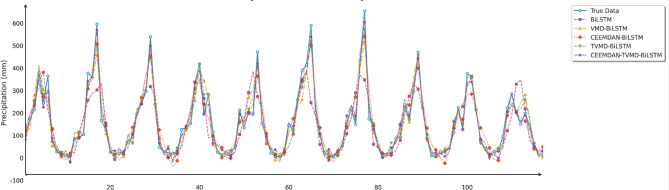
Line chart of predicted and observed values in Emeishan.

**Fig. 8:**
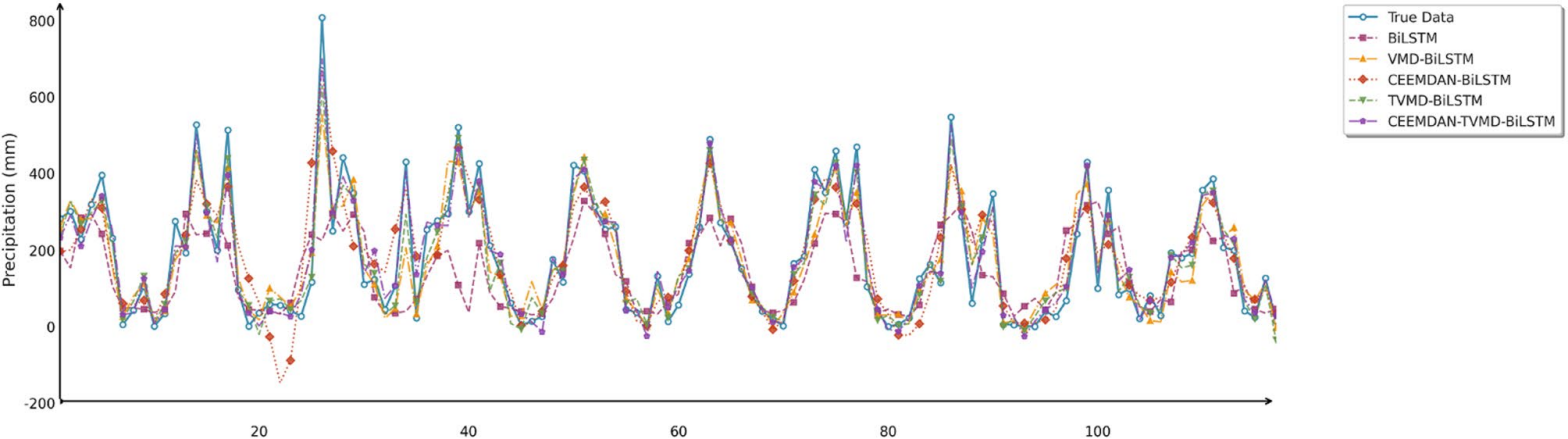
Line chart of predicted and observed values in Guangzhou.

**Fig. 9:**
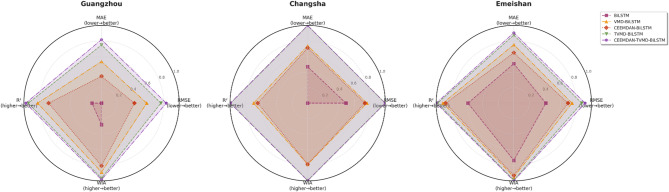
Multidimensional performance radar chart.

To comprehensively and intuitively compare the prediction errors of different models and evaluate the predictive performance of the models constructed in this study, a systematic assessment of five prediction models (BiLSTM, VMD-BiLSTM, CEEMDAN-BiLSTM, TVMD-BiLSTM, and CEEMDAN-TVMD-BiLSTM) across three different regions was conducted using seven charts. These charts included: scatter plots of predicted versus observed values for the three regions of Changsha, Emeishan, and Guangzhou (Figs. 3, 4 and 5), precipitation prediction time series comparison and absolute prediction error analysis charts for each region (Figs. 6, 7 and 8), and a multi-dimensional performance radar chart synthesizing all methods’ performance across the three cities (Fig. 9).

The scatter plot results demonstrated that compared to the single BiLSTM model, prediction models enhanced with data decomposition algorithms exhibited significant performance improvements, with the CEEMDAN-TVMD-BiLSTM and TVMD-BiLSTM models showing the most superior performance in terms of *R*² and consistency index, where data points were distributed more closely around the ideal diagonal line.

Several important phenomena were observed from the time series comparison charts. First, by comparing models employing decomposition algorithms (VMD-BiLSTM, TVMD-BiLSTM, CEEMDAN-BiLSTM, and CEEMDAN-TVMD-BiLSTM) with those without decomposition algorithms (BiLSTM), it was found that the BiLSTM model’s predictions exhibited pronounced lag phenomena, approximately equivalent to one prediction time interval. This lag phenomenon may be attributed to the insufficient feature extraction capability of the BiLSTM model for precipitation time series data, causing the model to tend to utilize data from the previous time step as the prediction for the current time step. In contrast, the prediction results from VMD-BiLSTM, TVMD-BiLSTM, CEEMDAN-BiLSTM, and CEEMDAN-TVMD-BiLSTM models did not exhibit such lag phenomena, indicating that decomposition algorithms can effectively decompose different dimensional feature fluctuation patterns in time series data, thereby improving feature extraction from each subsequence and enabling more accurate precipitation predictions. Second, when comparing VMD-BiLSTM and TVMD-BiLSTM models, it was clearly observed that the TVMD-BiLSTM model’s predictions were closer to actual observed values under both high and low precipitation conditions. This phenomenon indicated that TTAO could identify the most robust parameter combinations for precipitation time series data in different regions, thereby decomposing time series data into more accurate subsequences. Finally, through comparison of the prediction performance of CEEMDAN-BiLSTM, TVMD-BiLSTM, and CEEMDAN-TVMD-BiLSTM models, it was found that the secondary decomposition algorithm performed significantly better in data decomposition than the primary decomposition algorithm, further validating the effectiveness of multi-level decomposition strategies in improving prediction accuracy.

The multi-city dimensional performance radar chart revealed that the CEEMDAN-TVMD-BiLSTM model occupied the outer regions of the radar chart across most evaluation metrics, demonstrating its strong generalization capability and prediction accuracy under different geographical environments, thereby validating the effectiveness and superiority of the combined decomposition method proposed in this study.

### IPO optimization algorithm result

To verify the accuracy and feasibility of the IPO optimization algorithm proposed in this paper, this section conducted comparative experiments using the IPO-BiLSTM model, PO-BiLSTM model, and BiLSTM model in three typical cities. The prediction results of these models were shown in Table 4.

As can be seen from the comparison results of different models in Table 4, although manual setting of empirical parameters saves parameter optimization time, the PO and IPO optimization algorithms yield results that are more consistent with the actual conditions of each region. Furthermore, although the IPO constructed in this study requires more optimization time, the IPO optimization algorithm achieves more precise parameter optimization than the PO optimization algorithm, making it more suitable for practical applications.

**Table 4:** Prediction errors of different optimization algorithms.

City	Model	MSE	MAE	RMSE	*R* ^2^	I(WIA)	T(optimize)	T(train)	T(predict)
Changsha	BiLSTM	5545.786	54.253	74.470	0.247	0.624	-	32.582	0.007
	PO-BiLSTM	5512.968	53.389	74.249	0.251	0.610	43.967	18.899	0.008
	IPO-BiLSTM	5478.074	55.221	74.014	0.256	0.638	83.473	32.134	0.009
Emeishan	BiLSTM	7027.275	51.087	83.829	0.675	0.895	-	33.357	0.007
	PO-BiLSTM	6904.930	51.864	83.096	0.681	0.894	97.967	24.864	0.007
	IPO-BiLSTM	6937.411	52.141	83.291	0.679	0.899	160.002	31.296	0.007
Guangzhou	BiLSTM	17375.193	91.388	131.815	0.335	0.726	-	32.258	0.006
	PO-BiLSTM	17306.578	92.367	131.554	0.338	0.733	113.768	22.263	0.009
	IPO-BiLSTM	16969.852	91.412	130.268	0.351	0.734	962.439	35.110	0.008

**Fig. 10:**
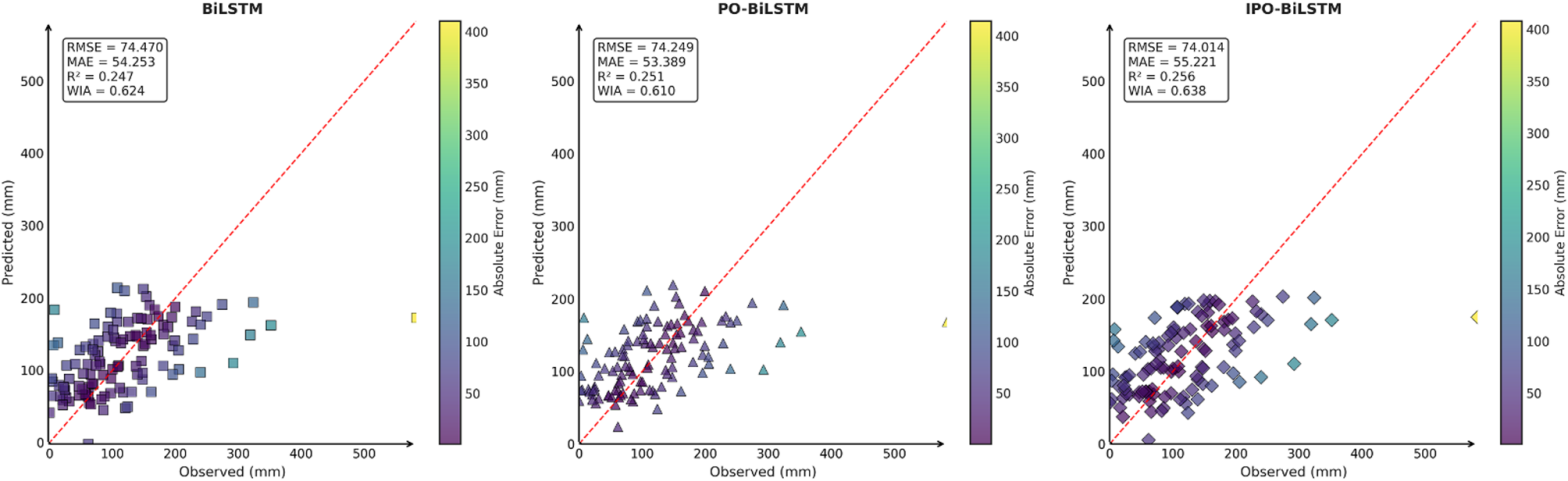
Scatter plot of predicted and observed values in Changsha.

**Fig. 11:**
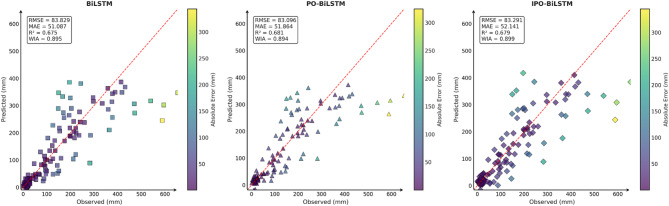
Scatter plot of predicted and observed values in Emeishan.

**Fig. 12:**
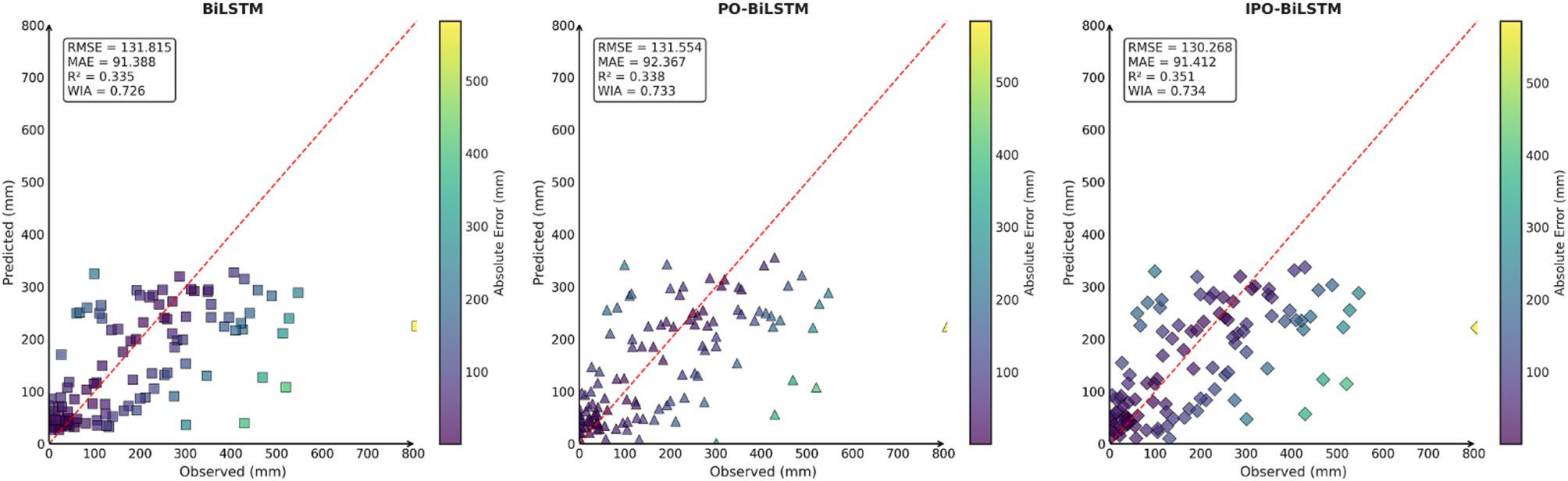
Scatter plot of predicted and observed values in Guangzhou.

**Fig. 13:**
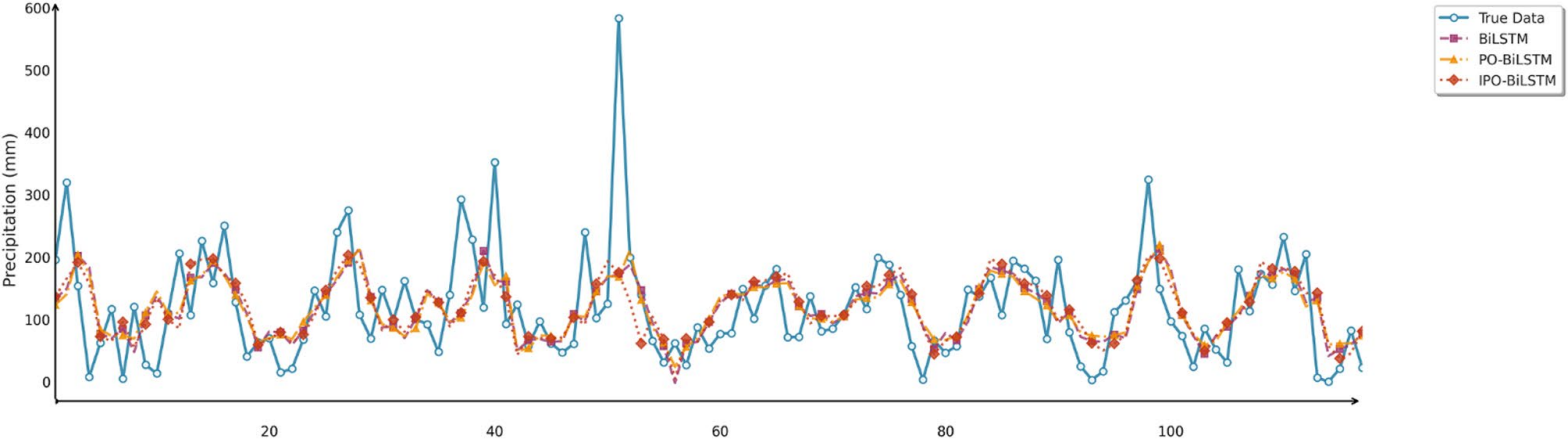
Line chart of predicted and observed values in Changsha.

**Fig. 14:**
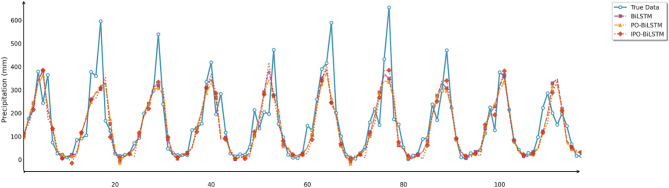
Line chart of predicted and observed values in Emeishan.

**Fig. 15:**
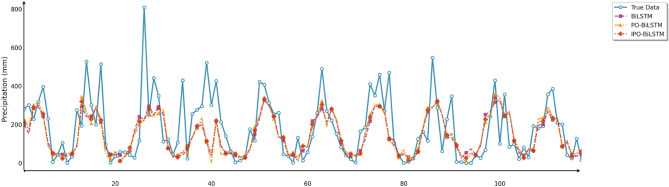
Line chart of predicted and observed values in Guangzhou.

**Fig. 16:**
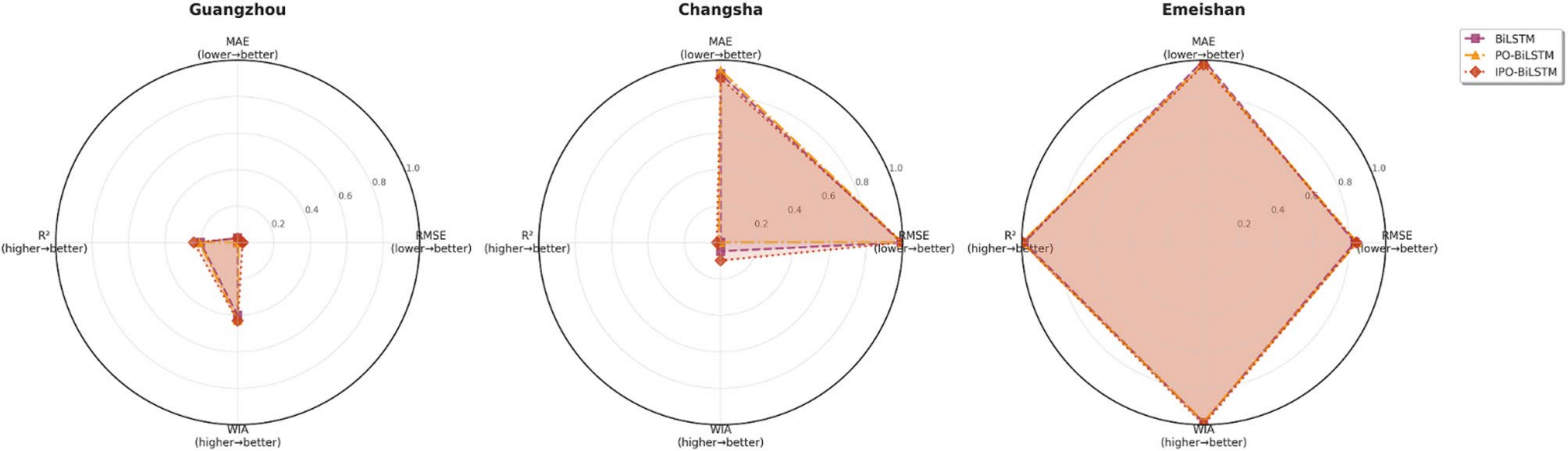
Multidimensional performance radar chart.

Similarly, this section employed seven charts to systematically evaluate three prediction models (BiLSTM, PO-BiLSTM, and IPO-BiLSTM) across three different regions. These charts also included: scatter plots of predicted versus observed values for the three regions of Changsha, Emeishan, and Guangzhou (Figs. 10, 11 and 12), precipitation prediction time series comparison and absolute prediction error analysis charts for each region (Figs. 13, 14 and 15), and a multi-dimensional performance radar chart synthesizing the performance of all methods across the three cities (Fig. 16).

The scatter plot analysis results demonstrated that compared to the baseline BiLSTM model, prediction models incorporating optimization algorithms exhibited moderate performance improvements. Across the Changsha, Emeishan, and Guangzhou regions, the IPO-BiLSTM model showed slight superiority over the PO-BiLSTM and BiLSTM models in terms of R² and consistency index, with data points distributed more closely around the ideal diagonal line.

The time series comparison charts revealed that regardless of whether optimization algorithms were employed, all models’ predictions exhibited pronounced lag phenomena, with lag times approximately equivalent to one prediction time interval. This further indicated that precipitation time series data contained multidimensional subsequences with different fluctuation patterns, rendering single models incapable of effectively extracting feature information from undecomposed data. Furthermore, comparison of the prediction performance between IPO-BiLSTM and PO-BiLSTM models clearly demonstrated that the IPO-BiLSTM model performed better in predicting sharp peaks. This suggested that the hyperparameters optimized by the IPO algorithm enhanced the robustness of the BiLSTM model, enabling it to better capture abrupt changes in precipitation data. Finally, while all optimization models displayed similar capabilities in tracking precipitation variation trends, IPO-BiLSTM exhibited higher stability in handling extreme value predictions.

The multi-city dimensional performance radar chart indicated that the IPO-BiLSTM model occupied relatively outer regional positions across most evaluation metrics, demonstrating that the IPO-BiLSTM model possessed relatively strong adaptability and prediction accuracy under different geographical environments. However, compared to models employing decomposition algorithms, the overall performance of all three models still required further improvement, thereby validating the crucial role of data decomposition techniques in precipitation prediction.

### Model prediction result

In order to fully verify the accuracy and feasibility of the prediction results of each module in the model constructed in this article, this section used CEEMDAN-TVMD-IPO-BiLSTM model, IPO-BiLSTM model, CEEMDAN-TVMD-BiLSTM model, and BiLSTM model to conduct comparative experiments in three typical cities. The model prediction results were shown in Table 5.

**Table 5:** Prediction errors of different models.

City	Model	MSE	MAE	RMSE	*R* ^2^	I(WIA)	T(optimize)	T(train)	T(predict)
Changsha	BiLSTM	5545.786	54.253	74.470	0.247	0.624	-	32.582	0.007
	CEEMDAN-TVMD-BiLSTM	236.572	12.298	15.381	0.968	0.991	-	473.128	0.091
	IPO-BiLSTM	5478.074	55.221	74.014	0.256	0.638	83.473	32.134	0.009
	CEEMDAN-TVMD-IPO-BiLSTM	208.672	11.804	14.445	0.972	0.992	2390.881	347.595	0.108
Emeishan	BiLSTM	7027.275	51.087	83.829	0.675	0.895	-	33.357	0.007
	CEEMDAN-TVMD-BiLSTM	619.635	19.599	24.892	0.971	0.992	-	634.697	0.122
	IPO-BiLSTM	6937.411	52.141	83.291	0.679	0.899	160.002	31.296	0.007
	CEEMDAN-TVMD-IPO-BiLSTM	503.861	17.329	22.447	0.977	0.994	2437.926	482.796	0.139
Guangzhou	BiLSTM	17375.193	91.388	131.815	0.335	0.726	-	32.258	0.006
	CEEMDAN-TVMD-BiLSTM	1208.064	26.606	34.757	0.954	0.987	-	536.747	0.110
	IPO-BiLSTM	16969.852	91.412	130.268	0.351	0.734	962.439	35.110	0.008
	CEEMDAN-TVMD-IPO-BiLSTM	1047.957	24.322	32.372	0.960	0.989	7523.378	451.538	0.128

From the comparison results of different model predictions in Table 5, it was evident that the CEEMDAN-TVMD-IPO-BiLSTM model proposed in this paper achieved the smallest prediction error across the three cities, with RMSE values of 32.373, 14.445, and 22.447, respectively. In contrast, the BiLSTM model had the largest prediction error, with RMSE values of 131.815, 74.470, and 83.829. Additionally, the CEEMDAN-TVMD-IPO-BiLSTM model achieves the highest *R*² values in the three cities, at 0.960, 0.972, and 0.977, respectively, whereas the BiLSTM model has the lowest *R*² values, at 0.335, 0.247, and 0.675. Comparing the CEEMDAN-TVMD-BiLSTM model and the CEEMDAN-TVMD-IPO-BiLSTM model constructed in this paper, it could be observed that the BiLSTM model optimized through the IPO algorithm effectively enhanced prediction accuracy, though the improvement was less pronounced than that achieved by the TVMD method. Furthermore, by comparing the IPO-BiLSTM model with the CEEMDAN-TVMD-IPO-BiLSTM model, it was apparent that the CEEMDAN-TVMD method could further improve model accuracy when applied to the BiLSTM model optimized by the IPO algorithm.

**Fig. 17:**
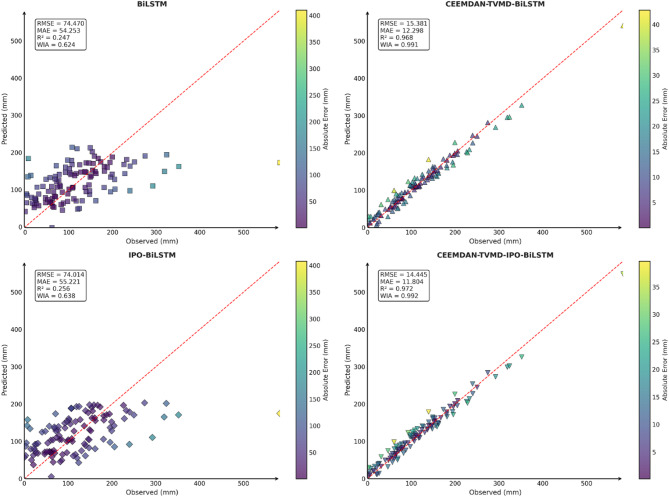
Scatter plot of predicted and observed values in Changsha.

**Fig. 18:**
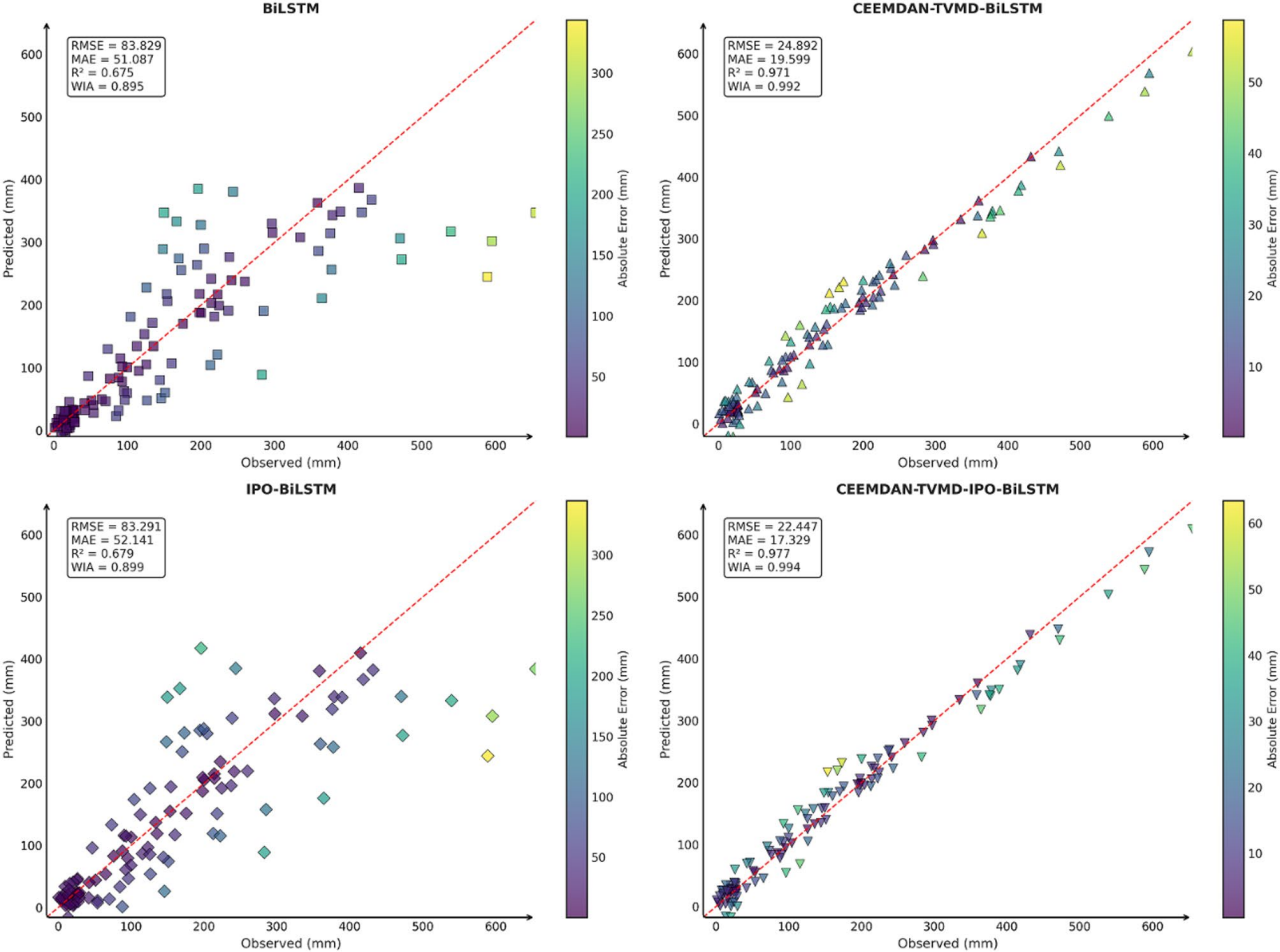
Scatter plot of predicted and observed values in Emeishan.

**Fig. 19:**
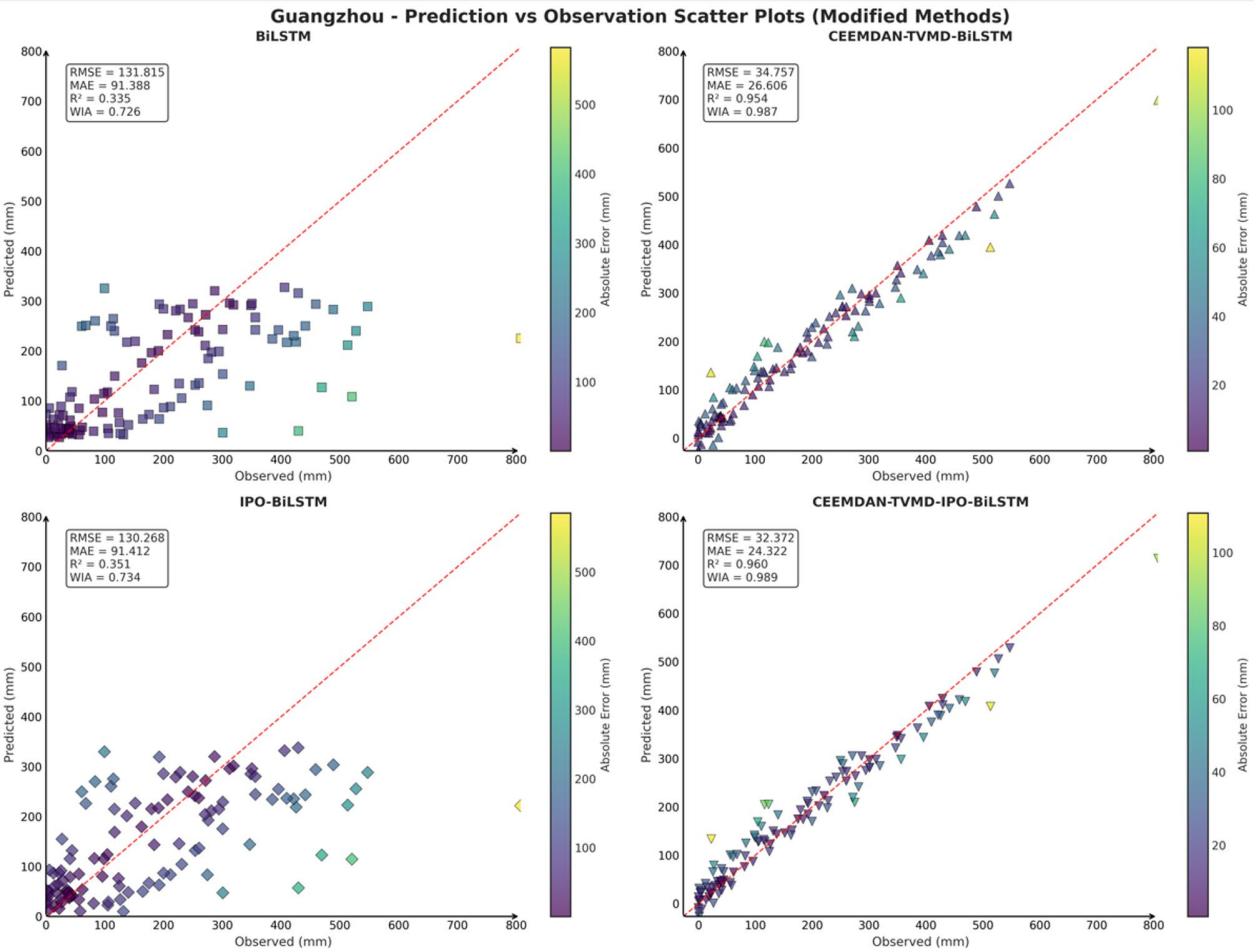
Scatter plot of predicted and observed values in Guangzhou.

**Fig. 20:**
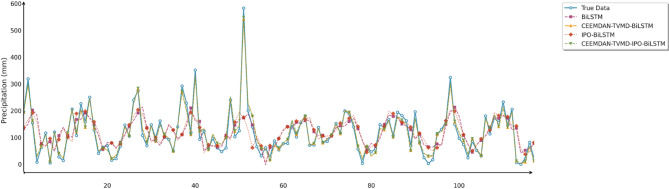
Line chart of predicted and observed values in Changsha.

**Fig. 21:**
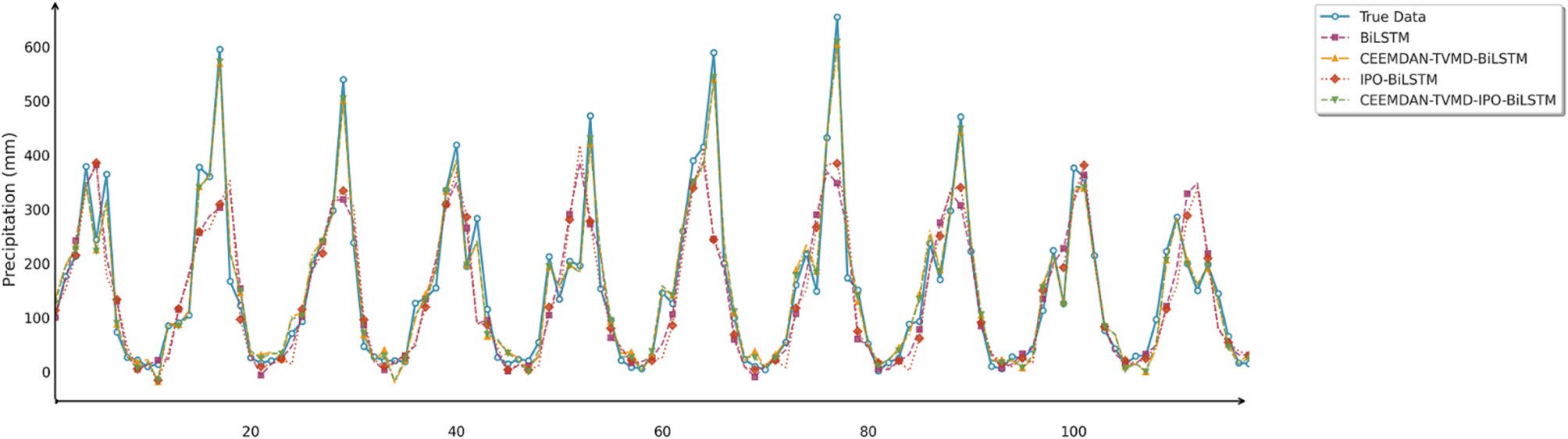
Line chart of predicted and observed values in Emeishan.

**Fig. 22:**
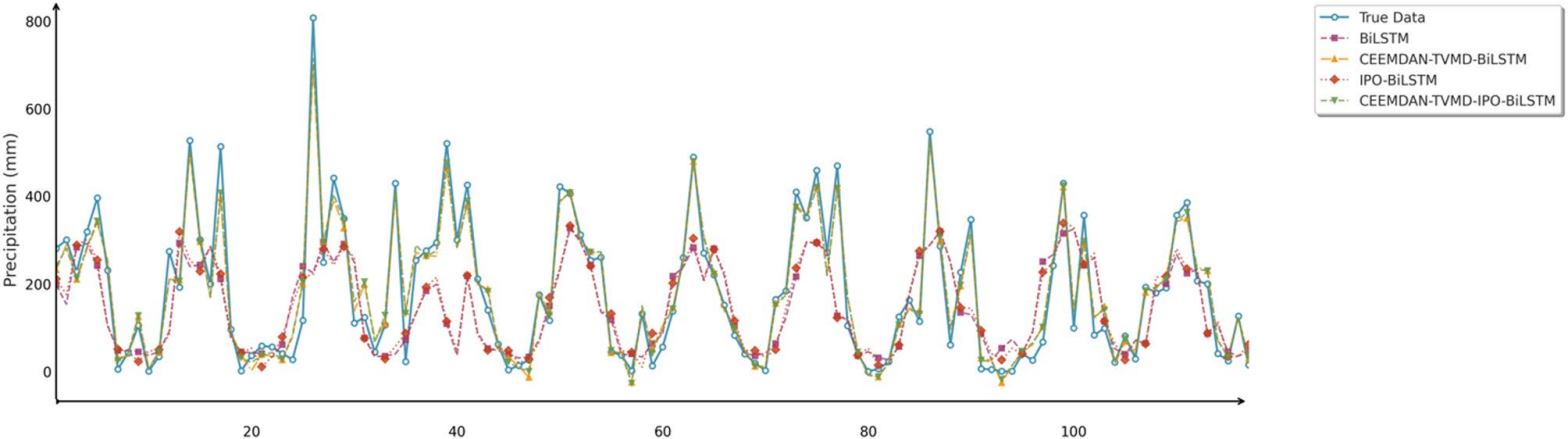
Line chart of predicted and observed values in Guangzhou.

**Fig. 23:**
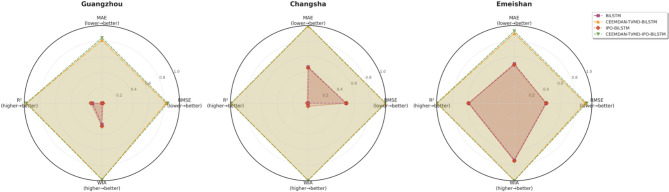
Multidimensional performance radar chart.

To comprehensively and intuitively compare the prediction errors of different models and evaluate the predictive performance of the models constructed in this study, this section similarly employed seven charts to systematically assess the ablation experimental results across three different regions, thereby demonstrating the collaborative optimization of multiple methods to improve precipitation prediction accuracy. These charts included: scatter plots of predicted versus observed values for the three regions of Changsha, Emeishan, and Guangzhou (Figs. 17, 18 and 19), precipitation prediction time series comparison and absolute prediction error analysis charts for each region (Figs. 20, 21 and 22), and a multi-dimensional performance radar chart synthesizing the performance of all methods across the three cities (Fig. 23).

From the comparison of predicted and observed values in the scatter plots, it can be seen that the CEEMDAN-TVMD-IPO-BiLSTM model performed most excellently, with its data points most closely distributed around the ideal diagonal line, demonstrating excellent prediction accuracy. This model achieved the highest correlation coefficients and consistency indices across all cities while maintaining the lowest error levels. In contrast, the basic BiLSTM model showed more scattered data point distribution with obviously insufficient prediction performance, while the other two improved models’ performance fell between the basic model and the optimal model.

The time series prediction comparison charts further revealed the differences in capabilities among various models in capturing dynamic precipitation changes. The CEEMDAN-TVMD-IPO-BiLSTM model demonstrated excellent peak-capturing capability when handling extreme precipitation events, accurately tracking the timing and intensity changes of sudden heavy precipitation. In contrast, the basic model often exhibited obvious lag phenomena and amplitude estimation deviations when facing complex precipitation patterns. During calm precipitation periods, the improved models showed higher stability, effectively suppressing noise interference and significantly reducing prediction deviations. Absolute error analysis results indicated that the basic BiLSTM model had the most severe error fluctuations, particularly during extreme weather events, while the combined optimization model consistently maintained stable and smaller error ranges.

From the performance radar chart, it can be seen that the CEEMDAN-TVMD-IPO-BiLSTM model formed the largest coverage area across all evaluation dimensions, fully demonstrating its superior performance under different geographical and climatic environments. The experimental results showed that the three cities exhibited regional characteristic differences in prediction difficulty, which mainly stemmed from their unique climate patterns and geographical conditions.

In summary, although the BiLSTM model had advantages in handling the nonlinear relationships of time series data, it showed significant biases in precipitation prediction, particularly in capturing complex precipitation patterns and noise processing. To overcome these limitations, the CEEMDAN-TVMD-BiLSTM model introduced the improved CEEMDAN-TVMD decomposition algorithm, which effectively removed noise and irregular fluctuations by decomposing the original precipitation time series into several intrinsic mode functions and residual components, thereby improving prediction accuracy. The CEEMDAN-TVMD-IPO-BiLSTM model combined the denoising and feature extraction advantages of CEEMDAN-TVMD with the parameter optimization of the BiLSTM model using the improved IPO, significantly enhancing parameter selection efficiency and model performance. As a result, the CEEMDAN-TVMD-IPO-BiLSTM model achieved the lowest errors on the test set and obtained the highest R² values and WIA indices, demonstrating its significant advantages in precipitation prediction.

### Parameter sensitivity analysis

In this parameter sensitivity analysis experiment, two core parameters of the IPO optimization algorithm were analyzed: population size and maximum number of iterations. These two parameters directly affect the search capability and convergence performance of the optimization algorithm, serving as key factors determining the optimization effectiveness. Among them, the population size determines the coverage extent of the search space during algorithm iterations, while the maximum number of iterations controls the search depth and convergence time of the optimization algorithm. Tables 6 and 7 present the parameter sensitivity experimental results for population size and maximum number of iterations across three cities, respectively.

**Table 6:** Sensitivity analysis of population size.

City	Population size	MSE	MAE	RMSE	*R* ^2^	I(WIA)	T(optimize)	T(train)	T(predict)
Changsha	1	246.330	12.714	15.695	0.967	0.991	427.469	387.317	0.107
	2	208.672	11.804	14.445	0.972	0.992	2390.881	347.595	0.108
	3	233.091	12.208	15.267	0.968	0.991	4316.575	395.292	0.116
	4	232.520	11.923	15.249	0.968	0.991	9057.798	437.816	0.111
Emeishan	1	559.641	18.208	23.657	0.974	0.993	995.460	520.088	0.137
	2	503.861	17.329	22.447	0.977	0.994	2437.926	482.796	0.139
	3	595.336	19.000	24.400	0.972	0.993	5778.264	547.729	0.131
	4	583.178	19.107	24.149	0.973	0.993	12137.521	521.091	0.138
Guangzhou	1	1185.787	26.058	34.435	0.955	0.987	803.960	423.790	0.116
	2	1105.096	25.041	33.243	0.958	0.988	2090.516	447.164	0.117
	3	1053.619	24.229	32.460	0.960	0.989	4999.453	446.153	0.129
	4	1120.842	24.818	33.479	0.957	0.988	9487.727	562.937	0.127

As shown in Table 6, the IPO-SVMD-BiLSTM model exhibits significant differences across the three cities under varying population sizes. Changsha and Emeishan achieve optimal performance with a population size of 2, while Guangzhou obtains the best results with a population size of 3. Furthermore, it can be observed that as the population size increases, the optimization time required by the algorithm grows exponentially, and the model’s prediction accuracy declines after reaching the optimal point as the population size continues to increase, indicating a clear performance saturation phenomenon in the model.

The differences in optimal population sizes mainly stem from the inherent complexity characteristics of precipitation data from different cities. As an eastern coastal city, Guangzhou exhibits more complex volatility and nonlinear features in its precipitation data, requiring a larger population size to adequately explore the solution space. In contrast, Changsha and Mount Emei, as central and western cities, have relatively simple precipitation data, allowing smaller populations to achieve effective search. When the population size is further increased, the predictive model performance of all three cities shows varying degrees of deterioration. This is primarily because excessive population size leads to reduced search efficiency, increases the risk of the algorithm becoming trapped in local optima, and significantly increases computational overhead.

**Table 7:** Sensitivity analysis of iteration number.

City	Iteration number	MSE	MAE	RMSE	*R* ^2^	I(WIA)	T(optimizate)	T(train)	T(predict)
Changsha	1	234.688	12.089	15.320	0.968	0.991	1010.810	396.104	0.118
	2	208.672	11.804	14.445	0.972	0.992	2390.881	347.595	0.108
	3	222.905	11.900	14.930	0.970	0.991	3229.813	308.094	0.108
	4	224.556	11.792	14.985	0.969	0.991	3327.745	345.794	0.110
Emeishan	1	543.986	18.278	23.324	0.975	0.993	1549.058	537.925	0.152
	2	503.861	17.329	22.447	0.977	0.994	2437.926	482.796	0.139
	3	548.835	18.615	23.427	0.975	0.993	4407.948	542.812	0.149
	4	559.119	18.275	23.646	0.974	0.993	6280.923	512.686	0.141
Guangzhou	1	1145.041	25.501	33.838	0.956	0.988	2760.610	459.420	0.119
	2	1053.619	24.229	32.460	0.960	0.989	4999.453	446.153	0.129
	3	1047.957	24.322	32.372	0.960	0.989	7523.378	451.538	0.128
	4	1088.460	24.846	32.992	0.958	0.988	11512.630	487.757	0.136

As shown in Table 7, the predictive models for Changsha and Mount Emei achieve optimal prediction results when the maximum number of iterations is 2, while Guangzhou requires the 3rd iteration to obtain optimal performance. This is also due to the fact that Guangzhou, as an eastern coastal city, has diversified meteorological driving mechanisms that make its precipitation patterns exhibit higher nonlinear characteristics and randomness, requiring a more thorough exploration process. The additional search time in the 3rd iteration helps the prediction algorithm better capture the intrinsic patterns of complex precipitation models. Similarly, all cities show performance degradation trends after exceeding the optimal number of iterations, which may be because the models overfitted the precipitation patterns in the training set while ignoring the inherent randomness and uncertainty of the climate system. Overly refined parameter adjustments may weaken the model’s generalization ability.

Therefore, from a practical application perspective, the above parameter sensitivity analysis can provide important theoretical guidance for the deployment of precipitation prediction models in different regions. For coastal cities with more complex climates, higher population sizes and maximum iteration numbers need to be set, with more computational resources invested for adequate hyperparameter optimization, while for inland regions with relatively simple climates, more economical parameter setting strategies can be adopted. This differentiated modeling approach not only improves prediction accuracy but also effectively controls computational costs, providing scientific basis for practical applications in meteorological forecasting services.

## Conclusion

Due to the randomness and uncertainty of monthly precipitation data, which can lead to insufficient extraction of time series features by models, this study aimed to effectively improve the accuracy of monthly precipitation prediction. Historical monthly precipitation data from three typical cities in China-an eastern city (Guangzhou), a central city (Changsha), and a western city (Emeishan)-were selected as research subjects. A CEEMDAN-TVMD-IPO-BiLSTM precipitation prediction model was constructed. The model first decomposed the original precipitation data using the CEEMDAN decomposition algorithm, output the modal components and residual components, and then used the topology optimization algorithm (TTAO) to optimize the VMD, and decomposed the high-dimensional sequence in the first decomposition result for the second time. Additionally, an IPO algorithm based on Cat and Cauchy-Gaussian variation was proposed to optimize the BiLSTM network. The optimized algorithm was then used to predict regional precipitation with a precisely constructed model. Experimental results indicatd the following: First, the proposed CEEMDAN-TVMD algorithm effectively captured nonlinear fluctuation patterns across different dimensions of the time series data, significantly improving the accuracy of the prediction model. Second, the IPO algorithm achieved more accurate hyperparameter tuning for the prediction model, thereby enhancing both prediction accuracy and model robustness. Compared to other models, the proposed CEEMDAN-TVMD-IPO-BiLSTM model achieved the lowest errors, the highest *R*² values, and the highest WIA, demonstrating its superiority in monthly precipitation prediction. Moreover, it better characterized the fluctuation patterns of precipitation, providing a scientific reference for formulating policies to mitigate drought and flood disasters.

Although the CEEMDAN-TVMD-IPO-BiLSTM model constructed in this study has achieved significant efficacy in monthly precipitation prediction, it also faces several challenges and limitations. First, the model integrates multiple complex algorithmic modules involving numerous hyperparameters that require selection and optimization. The optimal configuration of these parameters typically demands extensive empirical knowledge and experimental adjustments, thereby increasing the complexity and time costs of model implementation. Second, due to the adoption of a dual decomposition strategy, errors may propagate and accumulate across multiple decomposition and prediction stages. When prediction deviations occur at any stage, they may affect the stability and accuracy of the final reconstruction results. Finally, the model’s generalization capability is primarily based on validation from three Chinese cities, and its applicability under different climatic regions and meteorological conditions requires further verification. Future research can focus on the model’s adaptive parameter selection, computational efficiency optimization, cross-regional generalization capability enhancement, and improvement of prediction mechanism interpretability.

## Data Availability

The data are available from the first author on reasonable request.
